# Single-cell analysis of human embryos reveals diverse patterns of aneuploidy and mosaicism

**DOI:** 10.1101/gr.262774.120

**Published:** 2020-06

**Authors:** Margaret R. Starostik, Olukayode A. Sosina, Rajiv C. McCoy

**Affiliations:** 1 Department of Biology, Johns Hopkins University, Baltimore, Maryland 21218, USA;; 2 Department of Biostatistics, Johns Hopkins University, Baltimore, Maryland 21205, USA

## Abstract

Less than half of human zygotes survive to birth, primarily due to aneuploidies of meiotic or mitotic origin. Mitotic errors generate chromosomal mosaicism, defined by multiple cell lineages with distinct chromosome complements. The incidence and impacts of mosaicism in human embryos remain controversial, with most previous studies based on bulk DNA assays or comparisons of multiple biopsies of few embryonic cells. Single-cell genomic data provide an opportunity to quantify mosaicism on an embryo-wide scale. To this end, we extended an approach to infer aneuploidies based on dosage-associated changes in gene expression by integrating signatures of allelic imbalance. We applied this method to published single-cell RNA sequencing data from 74 human embryos, spanning the morula to blastocyst stages. Our analysis revealed widespread mosaic aneuploidies, with 59 of 74 (80%) embryos harboring at least one putative aneuploid cell (1% FDR). By clustering copy number calls, we reconstructed histories of chromosome segregation, inferring that 55 (74%) embryos possessed mitotic aneuploidies and 23 (31%) embryos possessed meiotic aneuploidies. We found no significant enrichment of aneuploid cells in the trophectoderm compared to the inner cell mass, although we do detect such enrichment in data from later postimplantation stages. Finally, we observed that aneuploid cells up-regulate immune response genes and down-regulate genes involved in proliferation, metabolism, and protein processing, consistent with stress responses documented in other stages and systems. Together, our work provides a high-resolution view of aneuploidy in preimplantation embryos, and supports the conclusion that low-level mosaicism is a common feature of early human development.

Genetic surveys of in vitro fertilized (IVF) human embryos consistently reveal substantial levels of aneuploidy—whole chromosome gains and losses that trace their origins to diverse mechanisms of chromosome mis-segregation. These include (primarily maternal) meiotic mechanisms such as nondisjunction, precocious separation of sister chromatids, and reverse segregation (Ottolini et al. 2015), as well as mitotic mechanisms such as mitotic nondisjunction, anaphase lag, and endoreplication (Vázquez-Diez and FitzHarris 2018). In contrast to meiotic errors, which uniformly affect all embryonic cells, mitotic errors generate chromosomal mosaicism, whereby different cells possess distinct chromosome complements. Such mitotic aneuploidies may propagate to descendant cells in a clonal manner and may also contribute to fitness variation. Although severe chromosomal mosaicism is lethal to early embryos (McCoy et al. 2015b; Ottolini et al. 2017), low levels of mosaicism appear compatible, and perhaps even common, with live birth (Greco et al. 2015; McCoy 2017).

One major limitation in studying the incidence and implications of chromosomal mosaicism is that most inferences are based on bulk DNA assays or comparisons of multiple biopsies of a few embryonic cells. As a result, current estimates of mosaicism in human embryos range from 4% to 90% (Capalbo et al. 2017). This has provoked intense debate over the true incidence of mosaicism at various developmental stages, its classification as a pathologic versus physiologic state, and its corresponding management in the context of preimplantation genetic testing for aneuploidy (PGT-A) of IVF embryos (Rosenwaks et al. 2018). Specifically, PGT-A seeks to prioritize IVF embryos for transfer based on the ploidy status of embryo biopsies, with current implementations involving biopsies of approximately five trophectoderm cells of Day-5 or Day-6 blastocysts. This approach is based on the premise that a biopsy is representative of the embryo as a whole and predictive of its developmental outcome. Although this premise may be violated by chromosomal mosaicism, the impact of such confounding remains obscure. A more complete picture of aneuploidy across many embryonic cells is therefore critical to a basic understanding of human development, as well as for guiding fertility applications such as PGT-A.

Single-cell genomic data sets offer promising resources for studying mosaic aneuploidy, as they potentially contain valuable information about both cell type and chromosome copy number. Moreover, characteristics of aneuploidies observed in single-cell data may suggest meiotic or mitotic mechanisms of origin. Previous studies have established proof-of-principle for detecting mosaic aneuploidy using single-cell RNA sequencing (scRNA-seq) data. Griffiths et al. (2017), for example, developed a statistical approach to discover aneuploidies based on chromosome dosage-induced changes in gene expression, validating their method using genome and transcriptome sequencing (G&T-seq) data (Macaulay et al. 2015). Other studies have developed similar approaches for the purpose of studying chromosome instability in cancer (Fan et al. 2018). In addition to changes in overall expression, aneuploidy is expected to generate allelic imbalance (i.e., allele-specific expression)—deviations from the null 1:1 ratio of expression from maternally and paternally inherited homologs. Here, we extended the expression-based method of Griffiths et al. (2017) to incorporate this complementary signature of allelic imbalance.

Applying this method to scRNA-seq data from 74 embryos (Petropoulos et al. 2016), we sought to quantify the incidence of meiotic and mitotic aneuploidy at single-cell resolution. Such knowledge is fundamental to uncovering downstream gene expression and fitness consequences of aneuploidy among the emerging cell lineages of the differentiating embryo. Together, our work provides an embryo-wide census of aneuploidy across early development and quantifies parameters of chromosomal mosaicism that have proven elusive to biopsy-based studies.

## Results

### Detection of aneuploidy in scRNA-seq data

**Figure 1.**
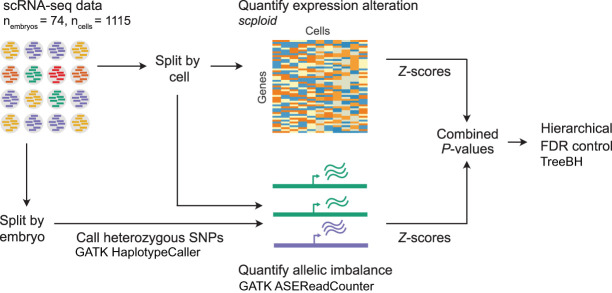
Approach for detecting aneuploidy in single-cell RNA-seq data based on complementary signatures of chromosome-wide gene expression alteration as well as allelic imbalance.

Building upon the foundations of Griffiths et al. (2017) (*scploid* software package), we developed an approach to integrate signatures of gene expression changes and allelic imbalance to discover aneuploidy in scRNA-seq data (Fig. 1; Methods). We assessed the value of this combined signature by simulating aneuploidies under a range of variance scenarios, finding that sensitivity was substantially improved without sacrificing (and often modestly improving) specificity compared to using the expression signature alone (Supplemental Figs. S1, S2; Supplemental Table S1).

Seeking to characterize aneuploidy at single-cell resolution throughout preimplantation development, we applied this approach to published scRNA-seq data from 88 human preimplantation embryos (1529 total cells) spanning the cleavage to late blastocyst stages (E3–E7) (Petropoulos et al. 2016). Cell type annotations were obtained from Stirparo et al. (2018) and, along with embryonic stage, were used to define strata for *scploid*. We removed cells in the lower 10th percentile of mapped reads or percent mapped reads, as well as two cell groups (E3 undifferentiated and E6 epiblast/primitive endoderm intermediate) that failed quality control due to their small numbers of sufficiently expressed genes (Supplemental Fig. S3). Exclusion of E3 cleavage-stage embryos may also be justified on the basis that the maternal-to-zygotic transition is not yet complete at this stage (Petropoulos et al. 2016). These quality control procedures resulted in the retention of 1115 cells from 74 embryos, spanning the E4 morula to E7 late blastocyst stages (Supplemental Table S2). Retained stages and cell types exhibited low expression variance, comparable to mouse embryo data used for benchmarking by Griffiths et al. (2017) (Supplemental Fig. S4). Evidence of aneuploidy based on signatures of expression alteration and allelic imbalance was correlated (Supplemental Fig. S5) but with the latter exhibiting greater sensitivity for detecting monosomy. We combined the two signatures using Fisher's method (Fisher 1925) to obtain *P*-values for every cell-chromosome combination (see Methods).

As highlighted in a previous review (Capalbo et al. 2017), the failure to account for multiple hypothesis testing has the potential to inflate estimates of mosaic aneuploidy based on multiple embryo biopsies. This challenge is magnified in single-cell analysis, where each cell-chromosome combination constitutes a separate statistical test (1115 cells × 22 autosomes = 24,530 tests in our study). Meanwhile, answering the most relevant biological questions requires integrating the output of many correlated statistical tests. For example, what proportion of embryos harbor at least one aneuploid cell? What proportion of cells within such embryos are aneuploid? We addressed this challenge using the method TreeBH (Bogomolov et al. 2017), an extension of the Benjamini–Hochberg procedure (Benjamini and Hochberg 1995) to tree-structured hypotheses. This allowed us to control the false discovery rate (FDR) at multiple levels (chromosomes, cells, and embryos) while accounting for the hierarchical dependency structure of the data.

**Figure 2.**
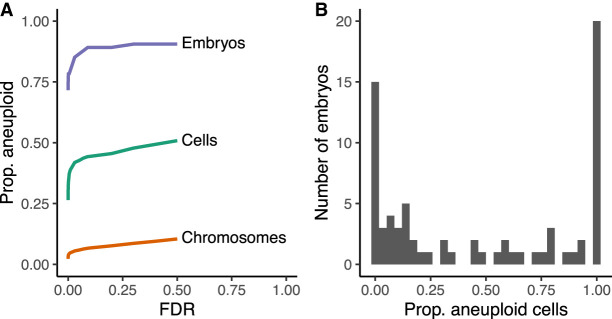
Aneuploidies discovered in scRNA-seq data from human preimplantation embryos (Petropoulos et al. 2016). (*A*) Proportions of aneuploid chromosomes, cells, and embryos detected at varying false discovery rates (FDR). Error rates were controlled while accounting for the hierarchical dependency structure of the data (chromosomes within cells within embryos) using TreeBH (Bogomolov et al. 2017). (*B*) Distribution of proportions of aneuploid cells per embryo at a 1% FDR.

**Figure 3.**
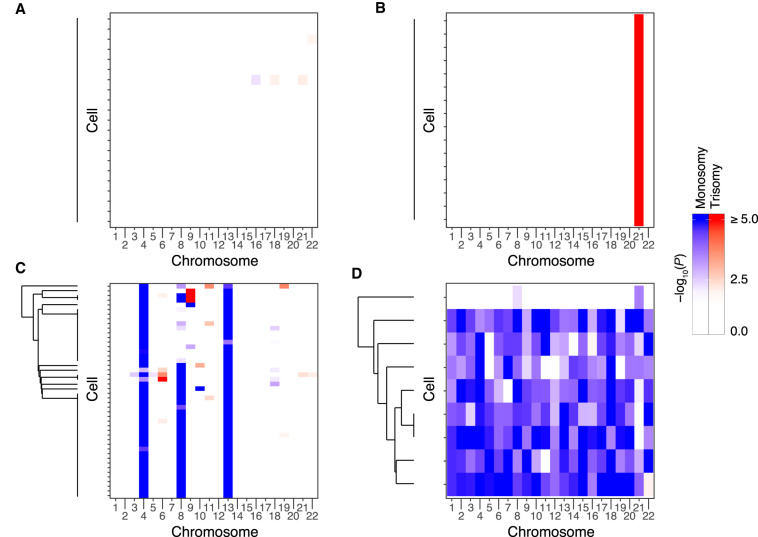
Examples of chromosome abnormalities detected based on scRNA-seq data from human embryos. Each heat map represents data from an individual embryo. Rows of the heat maps represent single cells, whereas columns represent chromosomes (autosomes only). Dendrograms depict hierarchical clustering of aneuploidy signatures, roughly reflecting common ancestry among aneuploid cells. (*A*) Embryo E7.3 was called euploid with negligible deviations from the null observed for all chromosomes within all cells. (*B*) Embryo E5.13 exhibits a putative meiotic-origin trisomy of Chromosome 21. (*C*) Embryo E7.17 exhibits putative meiotic-origin monosomies of Chromosomes 4 and 13, mosaic monosomy of Chromosome 8, and sporadic low-level aneuploidies of other chromosomes. (*D*) Embryo E7.5 was inferred as mosaic near-haploid, with haploid or near-haploid signatures in eight of nine cells, but near-diploidy in one cell.

Across all cell types and developmental stages, we estimated that 80% (59 of 74) of embryos contained at least one aneuploid cell and that 39% (433 of 1115) of all cells tested across the 74 embryos were aneuploid at an FDR threshold of 1% (Fig. 2A). A total of 4.8% (1172 of 24,530) of all cell-chromosome combinations were called as aneuploid. Patterns of aneuploidy across cells of individual embryos can help distinguish meiotic versus mitotic errors. Because they affect gametes and resulting zygotes, meiotic errors are expected to produce uniform aneuploidies across all embryonic cells. Mitotic aneuploidies meanwhile affect only a fraction of cells, depending upon the timing of their occurrence as well as the possibility of selection against aneuploid cells within mosaic embryos. We found that embryos displayed diverse patterns of aneuploidy, ranging from minor meiotic errors involving one or two chromosomes to chaotic mosaic abnormalities affecting many cells and chromosomes simultaneously (Figs. 2, 3; Supplemental Fig. S6). To allow for false negatives, we defined meiotic-origin aneuploidies using 75% as a heuristic cutoff for the percentage of cells per embryo with a particular aneuploidy (i.e., gain or loss of a particular chromosome). All other aneuploidies were classified as mitotic in origin. Based on these criteria, we observed that 5% (four of 74) embryos possessed only meiotic aneuploidies, 49% (36 of 74) of embryos possessed only mitotic aneuploidies, and 26% (19 of 74) of embryos possessed both meiotic and mitotic aneuploidies. An alternative meiotic error criterion defined as fewer than two normal cells of a given chromosome produced similar estimates (8% [six of 74 embryos] only meiotic, 51% [38 of 74 embryos] only mitotic, 22% [16 of 74 embryos] both meiotic and mitotic).

The proportion of aneuploid cells per embryo exhibited a characteristic “U”-shape, suggesting that, in addition to euploid embryos (e.g., Fig. 3A), meiotic aneuploidies (e.g., Fig. 3B) and low-level mosaic aneuploidies are relatively common but high-level mosaic aneuploidies are relatively rare (Fig. 2B). The paucity of high-level mosaic aneuploidies may reflect selection against such abnormalities prior to blastocyst formation—through embryonic mortality and/or exclusion of aneuploid cells (McCoy et al. 2015b; Bolton et al. 2016; Ottolini et al. 2017). Blastocyst E7.17 provides an example of multiple forms of aneuploidy within a single embryo (Fig. 3C). Specifically, Chromosomes 4 and 13 displayed evidence of meiotic origin monosomy, whereas Chromosome 8 displayed evidence of mosaic monosomy affecting approximately half of cells. The latter observation is potentially consistent with chromosome loss (e.g., via anaphase lag) during the first embryonic cleavage. Meanwhile, other chromosomes of this embryo displayed evidence of sporadic low-level aneuploidy. These include reciprocal monosomies and trisomies on Chromosomes 9 and 10, consistent with formation by mitotic nondisjunction. An even more extreme form of mosaicism was detected in blastocyst E7.5, which we inferred to be mosaic near-haploid (Fig. 3D). Eight of the nine cells showed chromosome-wide monoallelic expression, whereas one cell showed mostly biallelic expression. Due to its severe nature, this abnormality was only detectable based on signatures of allelic imbalance, as analysis based on overall expression alteration lacked a baseline for comparison (Supplemental Fig. S7). Allelic states of expressed SNPs in the near-haploid cells indicated a common parental origin of haploidy, as opposed to a mixture of haploid cells of distinct parental origins (Supplemental Fig. S8).

Although we observed a negative correlation between chromosome-specific aneuploidy rates and the number of protein-coding genes per chromosome (Pearson's *r* = −0.546, *P* = 8.64 × 10^−3^) (Supplemental Fig. S9), differences in aneuploidy rates among chromosomes were not significant upon accounting for nonindependence among chromosomes within cells within embryos (χ^2^[df = 21, *n* = 24,530] = 29.0, *P* = 0.114) (see Methods).

### No significant differences in aneuploidy rates among cell types of preimplantation embryos

Long-standing questions in the field of preimplantation genetics include how aneuploid cells are distributed among different cell types and how this changes throughout development. Cell-type-specific propensities and/or tolerances for aneuploidy could help explain observations such as confined placental mosaicism observed at later developmental stages (Toutain et al. 2018). Meanwhile, selection against aneuploid cells within mosaic embryos could help explain recent reports that some embryos that test mosaic with PGT-A can result in healthy live births after intrauterine transfer (Greco et al. 2015). Bolton et al. (2016) previously used a chimeric mouse model to address these questions, demonstrating that aneuploid cells of the inner cell mass undergo apoptosis, whereas aneuploid cells of the trophectoderm are tolerated but experience proliferative defects. Although groundbreaking, the relevance to human development has remained uncertain, as mouse embryos are known to exhibit lower rates of chromosome instability and higher rates of blastocyst formation than their human counterparts (Daughtry and Chavez 2016). Investigating these processes in human embryos is therefore essential for understanding the cellular and organismal fitness consequences of chromosomal mosaicism.

**Figure 4.**
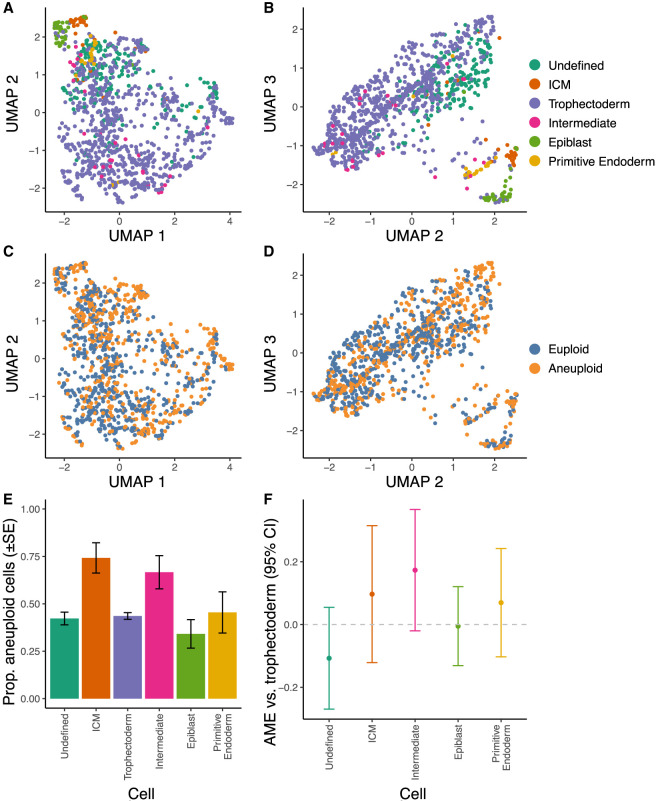
Comparisons of aneuploidy across cell types. (*A*) Individual cells plotted on the first and second UMAP dimensions, colored by cell type annotations from Stirparo et al. (2018). (*B*) Same as panel *A*, but for the second and third UMAP dimensions. (*C*) Cells plotted on the first and second UMAP dimensions, colored by aneuploidy status. (*D*) Same as panel *C*, but for the second and third UMAP dimensions. (*E*) Proportions of aneuploid cells, stratified by cell type. (*F*) Average marginal effects (AME) of cell types on aneuploidy rates relative to aneuploidy rates of trophectoderm cells—the source for PGT-A biopsies. Confidence intervals of all estimates overlap zero, indicating no significant difference for any cell type.

We obtained cell type annotations of the Petropoulos et al. (2016) data set from Stirparo et al. (2018) and confirmed that these groups formed clusters based on uniform manifold approximation and projection (UMAP) dimension reduction of the gene expression matrix (Fig. 4A,B). Cells did not noticeably cluster by aneuploidy status (Fig. 4C,D). We directly examined the relationship between aneuploidy and cell type using a binomial generalized linear mixed model (GLMM) with embryo as a random effect and embryonic stage (days post-fertilization) and cell type annotation as fixed effects (see Methods). We estimated the average marginal effect (AME) for a given predictor variable as its effect per cell, averaged across cells (see Methods). We compared this model to a reduced model without the cell type term to test whether aneuploidy rates varied across cell types. We detected no significant difference in aneuploidy rates across cell types (χ^2^[df = 5, *n* = 1115] = 6.19, *P* = 0.288) (Fig. 4E,F) or across days of development (E4 to E7; AME = −0.071, SE = 0.046, *P* = 0.124). We similarly detected no significant enrichment of aneuploidy in the trophectoderm versus the inner cell mass and its descendant lineages (AME = 0.012, SE = 0.039, *P* = 0.765). We note, however, that the wide confidence interval (95% CI [−0.064, 0.087]) signifies that we cannot rule out modest differences.

### Global gene expression responses to aneuploidy

In addition to the primary (i.e., *cis*-acting) and secondary (i.e., *trans*-acting) dosage effects, aneuploidy may induce tertiary transcriptional changes, including responses to proteotoxic, oxidative, and hypo-osmotic stresses (Dürrbaum et al. 2014; Tsai et al. 2019). To investigate this phenomenon in the context of human preimplantation development, we used a negative binomial mixed model to test for differential expression between euploid and aneuploid cells (see Methods). Embryo and cell type were specified as random effects to again account for the correlation among cells within embryos, whereas embryonic stage (days post-fertilization) and aneuploidy status were specified as fixed effects (see Methods).

**Figure 5.**
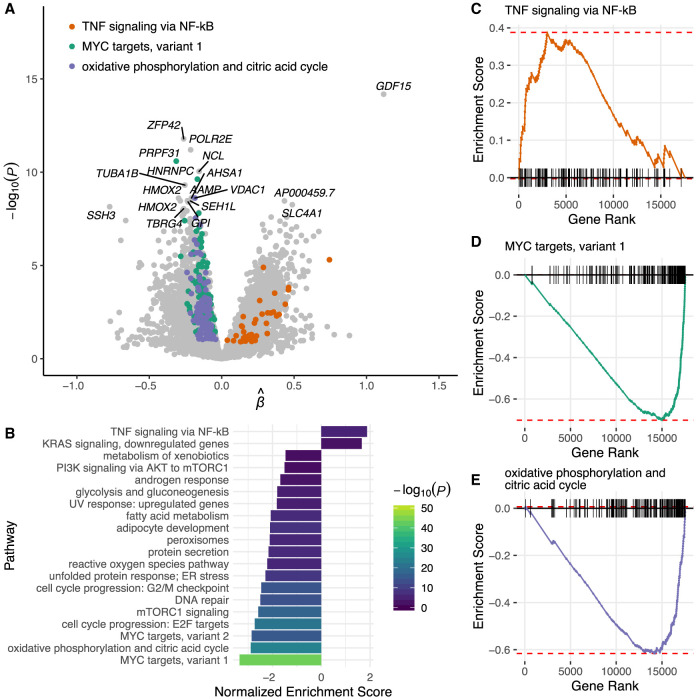
Transcriptional responses to aneuploidy in human embryos. (*A*) Volcano plot depicting differential expression between euploid and aneuploid cells. Positive values indicate increased expression in aneuploid cells, whereas negative values indicate reduced expression. (*B*) Hallmark gene sets from the Molecular Signatures Database (MSigDB) that are significantly enriched for genes that are up- or down-regulated in aneuploid cells based on gene set enrichment analysis (GSEA; 5% FDR). (*C*) Gene set enrichment plot demonstrating that genes regulated by NF-kB in response to tumor necrosis factor are significantly up-regulated in aneuploid cells. (*D*) Same as panel *C*, but demonstrating that MYC targets exhibit reduced expression in aneuploid cells. (*E*) Same as panel *C*, but demonstrating that genes involved in oxidative phosphorylation are down-regulated in aneuploid cells.

Using this model, we identified 2925 genes that were differentially expressed between euploid and aneuploid cells (5% FDR) (Fig. 5A; Supplemental Table S3). The most significant association involved up-regulation of growth differentiation factor 15 (*GDF15*) in aneuploid relative to euploid cells (β = 1.118, SE = 0.144, *P* = 6.6 × 10^−15^) (Supplemental Fig. S10). This gene was previously discovered to be up-regulated in aneuploid human cell lines compared to diploid cell lines from which they were derived, suggesting that *GDF15* may serve as a biomarker of aneuploidy across stages and cell types (Dürrbaum et al. 2014). Beyond this embryonic context, *GDF15* is well established as a cytokine that is up-regulated in response to cellular stress, with potential antiproliferative and anti-apoptotic functions (Kempf et al. 2006). The gene *ZFP42* exhibited the most significant down-regulation in aneuploid versus euploid cells (β = −0.262, SE = 0.037, *P* = 1.6 × 10^−12^) (Supplemental Fig. S11). This gene encodes a zinc finger protein that is a classic marker of pluripotency and whose expression contributes to lineage specification during early development (Son et al. 2013). The role of aneuploidy in altering such lineage decisions via *ZFP42* down-regulation may therefore merit future investigation.

We next tested for cell-type-specific gene expression responses to aneuploidy by fitting a separate model that included an interaction between cell type and aneuploidy status (see Methods). Such cell-type-specific responses may contribute to downstream heterogeneity in the developmental and fitness consequences of aneuploidy. We discovered 866 genes that exhibited significant aneuploidy × cell type interactions (5% FDR). The top such interaction involved the noncoding RNA *LINC00907*, which was strongly up-regulated in aneuploid blastomeres of undifferentiated cleavage-stage embryos (AME = 1.924, SE = 0.201, *P* = 1.1 × 10^−21^), in contrast to its negligible response to aneuploidy in various differentiated cell types (Supplemental Fig. S12). Other examples of interactions included the trophectoderm lineage marker *GATA3*, which exhibited up-regulation in undifferentiated aneuploid cells (AME = 0.818, SE = 0.239, *P* = 6.3 × 10^−4^) but down-regulation in aneuploid cells of the inner cell mass (AME = −0.940, SE = 0.326, *P* = 4.0 × 10^−3^) and descendant epiblast (AME = −0.973, SE = 0.249, *P* = 9.3 × 10^−5^) (Supplemental Fig. S12). Despite high baseline expression, *GATA3* exhibited no significant response to aneuploidy within the trophectoderm (AME = 0.023, SE = 0.077, *P* = 0.761) (Supplemental Fig. S12).

To gain further insight into global responses to aneuploidy in human embryos, we performed gene set enrichment analysis on the hallmark gene sets from the Molecular Signatures Database (MSigDB). This analysis revealed 20 gene sets that were significantly enriched among the tails of genes differentially expressed in aneuploid versus euploid cells (5% FDR) (Fig. 5B–E). We observed fewer gene sets significantly enriched for genes that are up- (two gene sets) versus down-regulated (18 gene sets) in aneuploid cells. Down-regulated gene sets included those related to cell proliferation, protein processing, and metabolism. Gene sets enriched for genes up-regulated in aneuploid cells included those that are down-regulated by KRAS signaling (again potentially reflecting an antiproliferation response) as well as genes regulated by NF-kB in response to TNF, broadly consistent with cellular stress and inflammatory signals previously reported in aneuploid cell lines (Liu et al. 2017; Santaguida et al. 2017).

### Aneuploidy calls based on scDNA-seq data corroborate our findings

Although informative of cell type, the sparse and bursty nature of scRNA-seq data poses a challenge for aneuploidy inference, placing practical limits on sensitivity and specificity (Griffiths et al. 2017). We thus sought to replicate the qualitative patterns of mosaic aneuploidy that we previously described using published data from additional disaggregated embryos that were analyzed by single-cell post-bisulfite adaptor tagging (PBAT) DNA methylome sequencing (Zhu et al. 2018). The fact that these data were based on single-cell DNA-sequencing (scDNA-seq) lends confidence to the aneuploidy calls. To facilitate comparison with our scRNA-seq results above, we focused on the 20 embryos from the morula and blastocyst stages of development. A total of 65% (13 of 20) of these embryos possessed at least one cell called as aneuploid. Applying the same definition we previously described (75% of cells of an embryo possessing a particular monosomy or trisomy), nine (45%) of these embryos possessed only mitotic aneuploidies, one (5%) embryo possessed only meiotic aneuploidies, and three (15%) embryos possessed both meiotic and mitotic aneuploidies. Hierarchical clustering of these aneuploidy calls revealed patterns qualitatively consistent with our scRNA-based results, including prevalent low-level mosaicism (Supplemental Fig. S13). Among the 12 blastocyst-stage embryos that could be tested, we detected no significant difference in the rates of aneuploidy between cells of the trophectoderm versus the inner cell mass (AME = 0.014, SE = 0.058, *P* = 0.811), again consistent with our scRNA-seq-based results.

### Cell-type-specific variation in aneuploidy may arise and intensify during postimplantation development

A recent study by Zhou et al. (2019) developed an extended in vitro culture system to produce the first single-cell genomic data from postimplantation human embryos spanning days 6–14 of development. This included Trio-seq data (including single-cell bisulfite sequencing) from 17 embryos, as well as scRNA-seq data from an additional 48 embryos. Applying the GLMM described above to published single-cell aneuploidy calls from these postimplantation embryos, we detected a significant enrichment of aneuploid cells in the trophectoderm compared to the epiblast and primitive endoderm (lineages derived from the inner cell mass). This enrichment was detected in scDNA-based calls from 286 cells of the 17 bisulfite-sequenced embryos (AME = 0.142, SE = 0.064, *P* = 0.028), as well as scRNA-based calls from 5911 cells of the 48 additional embryos (AME = 0.049, SE = 0.022, *P* = 0.024). Although we detected no overall change in per-cell aneuploidy rates across days 6 through 14 of development (AME = 0.021, SE = 0.012, *P* = 0.088), the scRNA-based calls revealed that the enrichment of aneuploid cells in the trophectoderm became stronger over time (β_lineage × stage_ = 0.212, SE = 0.068, *P* = 1.84 × 10^−3^). We note that this interaction model includes a random effect of embryo, thus addressing correlations among cells by allowing embryos to vary in their baseline rates of aneuploidy.

## Discussion

One key limitation of most previous studies of aneuploidy in human preimplantation embryos has been their reliance on biopsies of one or few cells. Many studies have adopted an operational definition of mosaicism based on PGT-A results that are intermediate between those expected of uniform euploid and uniform aneuploid biopsies (Cram et al. 2019). This narrow definition of mosaicism ignores the possibility of aneuploidy among the nonbiopsied cells that compose the rest of the embryo. In contrast, a biological definition of mosaicism denotes the presence of cells with distinct chromosome complements anywhere within the embryo. Although mathematical modeling approaches can help reconcile studies based on disparate definitions, such models require assumptions about unknown parameters including the spatial and lineage-specific distributions of aneuploid cells within mosaic embryos (Gleicher et al. 2017).

We sought to overcome these limitations by leveraging published scRNA-seq data from disaggregated human embryos. By combining signatures of gene expression alteration and allelic imbalance, we revealed patterns of meiotic and mitotic aneuploidy at single-cell resolution. A total of 31% of embryos displayed uniform or near-uniform aneuploidy of at least one chromosome across all cells—a pattern attributable to meiotic errors, which largely trace to maternal oogenesis. Meanwhile, low-level mosaicism was prevalent across all cell types and developmental stages, with 74% of embryos inferred to possess at least one cell affected by mitotic error. Although substantially higher than most biopsy-based studies, this estimate is roughly in line with the few previous studies to quantify aneuploidy in single cells of disaggregated embryos, albeit with different methodologies or at different stages. A landmark study by Vanneste et al. (2009) used SNP genotyping and array comparative genomic hybridization (CGH) to analyze 86 single cells from 23 disaggregated cleavage-stage embryos, finding that only three embryos were uniformly diploid, whereas the rest were either diploid-aneuploid mosaics or mosaics of entirely aneuploid cells. A recent study of 49 disaggregated cleavage-stage rhesus macaque embryos used scDNA-seq to demonstrate that 13 were euploid, nine were affected by solely meiotic errors, and the remaining 27 by mitotic errors or errors of ambiguous origin (Daughtry et al. 2019). Our estimates are also roughly consistent with aneuploidy calls based on scDNA- and scRNA-seq of human blastocysts (Zhu et al. 2018; Zhou et al. 2019), which reported evidence of mitotic-origin aneuploidy in more than half of embryos.

Our discoveries included one example of a mosaic near-haploid embryo (embryo E7.5) in which eight of nine cells appeared haploid or near-haploid, but one cell appeared near-diploid. This extreme form of mosaicism escaped detection based on gene expression analysis alone but was evident based on signatures of allelic imbalance. Hydatidiform moles are known to affect approximately one in 600 pregnancies, half of which are triploid dispermic (two paternal and one maternal set of chromosomes) and half of which are diploid androgenetic (two paternal sets of chromosomes). An estimated 85% of the latter type are monospermic and may arise via the extrusion of maternal chromosomes to the first polar body, followed by “diploidization” of the paternal chromosomes (Nguyen et al. 2018). The mosaic near-haploid constitution of embryo E7.5 is theoretically consistent with a dispermic origin, as a result of postzygotic diploidization of a triploid zygote (Golubovsky 2003). Uniparental diploidy is indistinguishable from haploidy with our approach. Fertilization with two sperm would explain the biallelic nature of the diploid cell, whereas the dispermic transmission of supernumerary centrioles could also induce mosaicism via multipolar mitosis. Although the IVF procedures used to produce the embryos sequenced by Petropoulos et al. (2016) were not reported, the growing use of intracytoplasmic sperm injection (ICSI) has reduced the prevalence of dispermy. Meanwhile, work in bovine embryos has revealed that even normally fertilized zygotes may produce mixoploid embryos by a mechanism termed “heterogoneic division” (Destouni et al. 2016). Specifically, chromosomes may segregate on an atypical gonomeric spindle to produce a mixture of androgenetic, gynogenetic, and normal diploid daughter cells, thus providing one alternative mechanism to explain embryo E7.5. We anticipate that future large-scale studies of disaggregated human embryos will reveal novel forms of mosaicism whose mechanisms of origin remain to be described.

By leveraging cell type information contained within scRNA-seq data, we also evaluated the long-standing question of how aneuploidy rates vary among cell types. Such comparisons provide insight into the developmental landscape of gene essentiality and dosage sensitivity, while also shedding light on the representativeness of PGT-A biopsies obtained from trophectoderm tissue. We detected no significant enrichment of aneuploidy within the trophectoderm but note that the wide confidence interval (AME = 0.012, 95% CI [−0.064, 0.087]) indicates that we cannot rule out modest differences. Indeed, the ∼6% enrichment of aneuploidy observed in trophectoderm cells of mature mouse blastocysts by Bolton et al. (2016) falls within this interval. Nevertheless, our study places bounds on any potential differences and provides a useful quantitative framework for testing such hypotheses in future single-cell data sets. Although we replicated this lack of enrichment using scDNA-seq-based calls from additional preimplantation embryos (Zhu et al. 2018), we detected significant enrichment of aneuploidy in the trophectoderm versus the primitive endoderm and epiblast (lineages derived from the inner cell mass) in published data from postimplantation embryos (Zhou et al. 2019). The latter data set included nearly 6000 cells, lending greater statistical power to such comparisons. The aneuploidy calls from Zhou et al. (2019) also revealed a significant interaction with day of development, indicating that the enrichment of aneuploidy in the trophectoderm becomes more extreme as development proceeds. Whether this observation reflects cell-type-specific apoptosis and/or proliferation defects of aneuploid cells is an open question for future study.

The transcriptional consequences of aneuploidy are known to extend beyond direct dosage effects to *trans*-acting impacts on other chromosomes as well as tertiary stress responses (FitzPatrick 2005; Sheltzer et al. 2012). Several recent studies have examined these responses using bulk RNA-seq analysis of embryos with specific aneuploidies (Kawai et al. 2018; Licciardi et al. 2018; Groff et al. 2019; Sanchez-Ribas et al. 2019; Weizman et al. 2019). However, bulk RNA-seq averages effects across cells, and studies of specific aneuploidies may conflate primary, secondary, and tertiary effects, thus hindering interpretation. We used a negative binomial mixed effects model to examine indirect responses to aneuploidy in single cells throughout early human development. This statistical method effectively accounts for nonindependence among cells within embryos. Such sampling designs are common among scRNA-seq data sets, and mixed effects models may be broadly applicable to differential expression analysis in this context. Our analysis revealed thousands of genes that are differentially expressed in aneuploid versus euploid cells. The top associated gene (*GDF15*) is a known biomarker of aneuploidy (Dürrbaum et al. 2014), thus supporting our approach. Although statistically robust, the distributions of *GDF15* expression in euploid and aneuploid cells overlapped substantially (Supplemental Fig. S10), underscoring the conclusion that no individual gene is diagnostic of aneuploidy status. Nevertheless, our results may be useful for exploring signatures of aneuploidy and associated stress responses, which could in turn be correlated with developmental outcomes. Indeed, multiple previous studies have suggested the utility of gene expression signatures for IVF embryo selection but have yet to be validated in large independent data sets (Vera-Rodriguez et al. 2015; Groff et al. 2019). In addition to main effects of aneuploidy on gene expression, we discovered hundreds of genes with significant cell-type-specific responses to aneuploidy. Among the top aneuploidy × cell type interactions was the transcription factor *GATA3*, a known trophectoderm lineage marker with a role in promoting trophectoderm fate (Home et al. 2017). *GATA3* was significantly up-regulated in undifferentiated aneuploid cells of cleavage-stage embryos. It is thus tempting to speculate that aneuploidy itself may bias lineage decisions, although our previous analysis suggests that any such biases are not sufficient to drive large differences in aneuploidy incidence among cell types.

A key methodological advance described here was the integration of signatures of expression alteration and allelic imbalance to detect aneuploidy in single cells. Consideration of allelic imbalance bolsters confidence in our results, especially in cases of monosomies, which generate monoallelic expression across entire chromosomes (allowing for technical artifacts such as barcode swapping, spurious SNPs, and mismapped reads). Despite this advance, inference of aneuploidy from scRNA-seq data remains challenging. One caveat is that any deviation of expression and allelic balance from diploid expectations could lead us to reject the null hypothesis, whereas phenomena other than whole-chromosome aneuploidy may occasionally induce such deviations. For example, large structural variation may be falsely classified as aneuploidy by our approach. Although the effect size thresholds that we implemented help mitigate this concern, approaches to explicitly distinguish segmental and whole-chromosome aneuploidies are priorities for future development. Additional opportunities for methodological improvement include the integration of population-based and/or read-based phasing into allelic imbalance analysis, which could be especially beneficial for the inference of trisomies (Loh et al. 2018).

The Petropoulos et al. (2016) data set used in our study was generated using the Smart-Seq2 platform, which captures full-length transcripts, albeit with 3′ bias. This contrasts with recent droplet-based platforms such as Chromium (10x Genomics) that achieve higher throughput (i.e., number of cells) but are limited to 3′ end sequences upstream of poly(A) tails (Haque et al. 2017). The full transcript coverage of Smart-Seq2 is advantageous for our application, as it enhances SNP discovery and enables quantification of allelic imbalance. Smart-Seq2 and other plate-based protocols also generally achieve higher capture efficiency than droplet-based platforms, which is important for low input applications such as sequencing of embryos composed of few cells. We anticipate that future improvements in the sensitivity and precision of scRNA-seq platforms will enhance the downstream accuracy of aneuploidy detection. Moreover, application of methods such as G&T-seq (Macaulay et al. 2015) to human embryos will combine the accuracy of aneuploidy detection using scDNA-seq with the rich biological insights enabled by scRNA-seq.

One additional caveat is that the data analyzed in our study derive from IVF embryos obtained from relatively few patients, about whom no demographic or clinical information was published. We therefore urge caution in extrapolating these findings to a broader population. Previous studies have established a strong association between maternal age and incidence of meiotic error in preimplantation embryos (Hassold and Hunt 2001). Studies have also revealed significant, albeit modest, associations between aneuploidy rates and various fertility diagnoses (McCoy et al. 2015b; Kort et al. 2018), as well as patient genotypes (McCoy et al. 2015a; Chernus et al. 2019). One persistent concern with all studies of preimplantation embryos is the possibility that IVF culture conditions impact chromosome stability. Indeed, such impacts have been documented by comparing in vitro versus in vivo matured bovine embryos (Tšuiko et al. 2017), although no such differences have been detected in humans during preimplantation development (Munné et al. 2020) or at live birth (Zamani Esteki et al. 2019).

Aneuploidy is the leading cause of pregnancy loss and congenital birth defects in humans (Hassold and Hunt 2001). As genetic testing platforms have improved, the existence of chromosomal mosaicism is increasingly acknowledged, but the prevalence of this phenomenon remains disputed, and the impacts on human development remain unclear (McCoy 2017). Here, we developed an approach to use scRNA-seq data from disaggregated human embryos to quantify aneuploidy and mosaicism at single-cell resolution. Our results support the conclusion that meiotic aneuploidies and low-level mosaic aneuploidies are common, but high-level mosaic aneuploidies are relatively rare. Aneuploidy rates among various cell types are similar during preimplantation development but may arise and intensify throughout postimplantation development. Together, our study reconciles disparate estimates of mosaicism based on different definitions and provides a quantitative framework for investigating aneuploidy in ever-growing single-cell data sets.

## Methods

### Aneuploidy inference on scRNA-seq data

Aneuploidy inference was based on complementary signatures of chromosome-wide differential expression and allelic imbalance. The differential expression signature is the basis of the software package *scploid* (Griffiths et al. 2017), which was previously benchmarked using genome and transcriptome sequencing data from mosaic aneuploid mouse embryos.

Gene expression quantifications from the Petropoulos et al. (2016) data set were obtained from conquer (Soneson and Robinson 2018) as input to *scploid*. Analysis was limited to the autosomes, as required by the software. Cells in the lower 0.1 quantile of mapped reads and/or percent mapped reads were excluded from analysis. Cell type annotations were obtained from Stirparo et al. (2018) and visualized on both principal component (Supplemental Fig. S3A) and UMAP (Fig. 4A,B) dimensions using Monocle (Trapnell et al. 2014) to confirm their clustering. Embryonic stage and cell type annotations were used to define strata as input to *scploid*, thereby limiting cell-type- and stage-specific variation that may confound aneuploidy inference. Groups that failed *scploid* quality control procedures were dropped from the analysis (Supplemental Fig. S3B). This resulted in the inclusion of 1115 cells from 74 embryos across 11 stage/cell type groups. Each group expressed between 3053 and 3351 genes at a median of 50 counts per million reads mapped (CPM) or greater per cell.

### Allelic imbalance Z-scores

Raw single-cell RNA-seq data from Petropoulos et al. (2016) were obtained from EMBL-EBI ArrayExpress (E-MTAB-3929). Reads were mapped to the reference genome using STAR (v2.7.1a) (Dobin et al. 2013). Single-cell alignments from the same embryo were then merged using SAMtools (v1.9) (Li et al. 2009) and processed for variant discovery with GATK (v4.0.12.0) (McKenna et al. 2010), according to the following workflow: https://gatkforums.broadinstitute.org/gatk/discussion/3891/calling-variants-in-rnaseq. Using embryo-level heterozygous SNPs and corresponding single-cell alignments as input, allelic read counts were then computed at every heterozygous SNP in every cell using the ASEReadCounter tool of GATK (Castel et al. 2015). The minimum of the counts of reference and alternative allele-supporting reads was obtained for every heterozygous SNP, summed across the chromosome, then divided by the total read count for that chromosome to obtain an allelic imbalance ratio.

To convert the observed allelic imbalance ratios to *Z*-scores, we first regressed out the effect of the number of reads on allelic imbalance since we observed a positive correlation between the observed proportions and the number of reads used to generate them. Then, under the assumption that most cell-chromosome combinations would lie under the null, we identified all null data points as those whose allelic imbalance estimates lie between the first quartile and the third quartile of the empirical distribution. We then estimated the residual at the null proportion as the mean of all the generated residuals for the null data points. To estimate the variance under the null, we further assumed that the residuals were approximately normally distributed. With this assumption and recalling that we already assumed that most cell-chromosome combinations would lie under the null, we derived the variance of the residual of the allelic imbalance under the null using the formula below:

$$\sigma^{2}\;=\;{\left(\frac{IQR}{2^{*}\;\Phi^{-1}(0.75)}\right)}^{2},$$

where IQR is the empirical interquartile range of the residuals, and Φ^−1^ is the inverse of the standard normal cumulative distribution function. This is motivated by the fact that under the normal distribution, the interval, I, contains the middle 50% of the data as

$$I\;=\;\mu \;\pm \;\sigma^{*}\;\Phi^{-1}(0.75).$$

This interval has length

$$L\;=\;2^{*}\;\sigma^{*}\;\Phi^{-1}(0.75)\approx IQR.$$

Hence, solving for σ and squaring it, we obtain the result above. With the mean and variance obtained under the null, we then converted the residuals to *Z*-scores by subtracting this mean from each point and dividing by the square root of the variance. We then computed one-sided *P*-values, based on the expectation that both monosomy and trisomy will have the effect of increasing allelic imbalance.

### Omnibus test combining allelic imbalance and *scploid* results

We conducted an omnibus test for each cell-chromosome using the *P*-values obtained from the *scploid* and allelic imbalance analyses described above. We note that, as a last step, *scploid* imposes an effect size threshold (|1−*s*_*ij*_| ≥0.2) to classify a chromosome as aneuploid (Griffiths et al. 2017). To incorporate this threshold into our analysis, we set *scploid P*-values for cell-chromosomes below this threshold to 1. We combined allelic imbalance and *scploid P*-values using Fisher's method (Fisher 1925). Correction for multiple testing was then carried out using TreeBH (Bogomolov et al. 2017) to account for the hierarchical nature of the data. Hypothesis tree structure was defined as chromosomes nested within cells nested within embryos, and FDR was controlled at 1% at each level. To assign aneuploid chromosomes to the categories of monosomy and trisomy, we applied *k*-means clustering (*k* = 2) to the *Z*-scores from the allelic imbalance and *scploid* analyses. Cell-chromosomes composing the cluster with lower mean *Z*-scores were classified as monosomic, and cell-chromosomes composing the other cluster were classified as trisomic.

### Simulation studies

We performed simulations to investigate the impact of integrating signatures of allelic imbalance with signatures of overall gene expression alteration. We generated data using a previously described simulation framework (Griffiths et al. 2017) with modifications to extend the simulation to allelic imbalance data. Specifically, we used our previously inferred ploidy states for each cell-chromosome combination to simulate new gene expression counts and allelic imbalance ratios expected given those ploidy states. Allelic ratios were randomly drawn from beta distributions whose mean and variance parameters were inferred from the data, stratified by inferred ploidy state. We then inferred the ploidy state of each simulated cell-chromosome combination using the gene expression signature alone, as well as using the combined gene expression and allelic imbalance signatures, integrated using the aforementioned omnibus test. By comparing inferred aneuploidies to the truth set from which we simulated, we could then test whether integrating allelic imbalance information provided any improvement relative to using the expression signature alone. In addition to simulating data using variance parameters for gene expression and allelic imbalance informed by the Petropoulos et al. (2016) data, we also evaluated performance under scenarios of reduced or inflated variance. We implemented this scaling by multiplying the baseline variance of each signature by an overdispersion factor, set to 0.3, 1, and 5. We repeated our simulations 100 times for each combination of overdispersion parameters and calculated mean sensitivity, specificity, and other performance metrics reported in Supplemental Figures S1 and S2 and Supplemental Table S1.

### Cell-type-specific propensity for aneuploidy

We assessed the effect of cell type on aneuploidy status using mixed-effects logistic regression. We applied these models to our own aneuploidy calls based on the Petropoulos et al. (2016) scRNA-seq data, as well as aneuploidy calls based on single-cell DNA- and RNA-sequencing of additional embryos, obtained from supplemental tables of Zhu et al. (2018) and Zhou et al. (2019). We fit two models to each data set. In both models, our outcome was a binary variable indicating aneuploidy status (defined as aneuploid if the cell possessed one or more aneuploid chromosome) and embryonic stage (days postfertilization), which was treated as a fixed effect continuous variable. In the first model, we included cell type (categorical variable), and in the second model, we included an indicator variable for the trophectoderm cells (with all other cell types grouped as nontrophectoderm). We treated embryo as a random effect to account for correlation among cells within embryos and estimated the random intercept for both models. Embryos were meanwhile assumed independent of one another.

### Average marginal effects

Let *E*(*Y*_*ij*_| *X*_*i*_, *b*_*j*_) represent the fitted values from each of the generalized linear mixed models defined above for the *i*th cell with the *j*th random effect (e.g., in the cell-type-specific propensity analysis, *j* refers to the *j*th embryo) and *X* is the covariate whose AME we wish to calculate. The AME is defined for a binary covariate *X* as

$$\frac{1}{n_{1}}\overset{n}{\underset{i}{\sum }}I(X_{i}\;=\;1)E(Y_{ij}|\;X_{i},\;b_{j})\;-\;\frac{1}{n_{2}}\overset{n}{\underset{i}{\sum }}I(X_{i}\;=\;0)E(Y_{ij}|\;X_{i},\;b_{j}),$$

where $n_{1}=\sum_{i}^{n}I(X_{i}\;=\;1)\;$ and $n_{2}=\sum_{i}^{n}I(X_{i}\;=\;0)$. We estimated these values and their standard errors using the *margins* package in R (https://CRAN.R-project.org/package=margins).

### Differential expression and gene set enrichment analysis

Global transcriptional responses to aneuploidy were investigated by testing for differential expression between cells called as euploid versus aneuploid. Single-cell expression counts were normalized using SCnorm (Bacher et al. 2017), with cell type annotations specified as biological conditions. Analysis was limited to broadly expressed genes with ≥1 normalized expression count for at least half of the cells. To mitigate direct dosage (i.e., *cis*-acting) effects of aneuploidy, we restricted each test to cells called as euploid for the chromosome containing the respective gene. For each gene, we then fit a negative binomial mixed model, implemented with lme4 (Bates et al. 2015). Normalized read counts (plus a pseudocount) were specified as the response variable, cell type and embryo were specified as crossed random effects, and embryonic stage (i.e., day postfertilization) and aneuploidy status were specified as fixed effects. We fit both a random slope and intercept, as well as a random intercept-only model for the cell type variable, retaining the more complex model only if it significantly improved fit over the reduced model based on analysis of deviance (α = 0.05). Coefficients, test statistics, and *P*-values were evaluated for the aneuploidy status term. Models producing convergence warnings (2.1% or 383 of 17,970 genes) were dropped from the analysis.

To identify genes with cell-type-specific differences in gene expression between euploid and aneuploid cells, we added an interaction term to our previous model. Our outcome variable was the normalized read counts of a given gene across cells. We assume a negative binomial distribution for the counts, to account for possible overdispersion in our data, and fit a generalized linear mixed model with embryo-specific random intercepts and fixed independent variables consisting of (1) the aneuploidy status (defined as aneuploid if the cell possessed one or more aneuploid chromosome), (2) embryonic stage (days postfertilization), treated as a fixed effect continuous variable, and (3) the cell type (epiblast, inner cell mass, intermediate, primitive endoderm, trophectoderm, or undifferentiated), which was treated as a categorical variable. In addition, we added an interaction term between aneuploidy status and each cell type to obtain cell-type-specific estimates of differential expression between euploid and aneuploid cells. We accounted for correlation among cells within embryos by treating embryo as a random effect. For each gene, we used a likelihood ratio test to evaluate the joint effect of all interaction terms in our model. For genes where our likelihood ratio tests were significant, we ran additional analyses to estimate the cell-type-specific AMEs.

Gene set enrichment analysis (Subramanian et al. 2005) was performed using the FGSEA (Korotkevich et al. 2019) package in R (R Core Team 2019). In order to limit the number of tests and improve biological interpretability, we focused our analysis on the Molecular Signatures Database hallmark gene sets (Liberzon et al. 2015), accessed via msigdbr (https://CRAN.R-project.org/package=msigdbr). Genes were ranked by signed *P*-value as input to GSEA, which was run using the adaptive multilevel splitting option to compute arbitrarily small *P*-values.

### Software availability

All codes necessary for reproducing our analyses are available at GitHub (https://github.com/mccoy-lab/aneuploidy_scrnaseq) and Zenodo (DOI: 10.5281/zenodo.3790930) and also provided as Supplemental Code.

## Competing interest statement

The authors declare no competing interests.

## Supplementary Material

Supplemental Material

## References

[GR262774STAC1] Bacher R, Chu L-F, Leng N, Gasch AP, Thomson JA, Stewart RM, Newton M, Kendziorski C. 2017 SCnorm: robust normalization of single-cell RNA-seq data. Nat Methods 14: 584–586. 10.1038/nmeth.4263 28418000 PMC5473255

[GR262774STAC2] Bates D, Mächler M, Bolker B, Walker S. 2015 Fitting linear mixed-effects models using lme4. J Stat Softw 67: 1–48. 10.18637/jss.v067.i01

[GR262774STAC3] Benjamini Y, Hochberg Y. 1995 Controlling the false discovery rate: a practical and powerful approach to multiple testing. J Royal Stat Soc Ser B Methodol 57: 289–300. 10.1111/j.2517-6161.1995.tb02031.x

[GR262774STAC4] Bogomolov M, Peterson CB, Benjamini Y, Sabatti C. 2017 Testing hypotheses on a tree: new error rates and controlling strategies. *arXiv*:1705.07529v2 [stat.ME]. 10.1093/biomet/asaa086 PMC9945647 36825068

[GR262774STAC5] Bolton H, Graham SJL, Van der Aa N, Kumar P, Theunis K, Fernandez Gallardo E, Voet T, Zernicka-Goetz M. 2016 Mouse model of chromosome mosaicism reveals lineage-specific depletion of aneuploid cells and normal developmental potential. Nat Commun 7: 11165 10.1038/ncomms11165 27021558 PMC4820631

[GR262774STAC6] Capalbo A, Ubaldi FM, Rienzi L, Scott R, Treff N. 2017 Detecting mosaicism in trophectoderm biopsies: current challenges and future possibilities. Hum Reprod 32: 492–498. 10.1093/humrep/dew250 27738115 PMC5400043

[GR262774STAC7] Castel SE, Levy-Moonshine A, Mohammadi P, Banks E, Lappalainen T. 2015 Tools and best practices for data processing in allelic expression analysis. Genome Biol 16: 195 10.1186/s13059-015-0762-6 26381377 PMC4574606

[GR262774STAC8] Chernus JM, Allen EG, Zeng Z, Hoffman ER, Hassold TJ, Feingold E, Sherman SL. 2019 A candidate gene analysis and GWAS for genes associated with maternal nondisjunction of chromosome 21. PLoS Genet 15: e1008414 10.1371/journal.pgen.1008414 31830031 PMC6932832

[GR262774STAC9] Cram DS, Leigh D, Handyside A, Rechitsky L, Xu K, Harton G, Grifo J, Rubio C, Fragouli E, Kahraman S, 2019 PGDIS position statement on the transfer of mosaic embryos 2019. Reprod Biomed Online 39: e1–e4. 10.1016/j.rbmo.2019.06.012 31421710

[GR262774STAC10] Daughtry BL, Chavez SL. 2016 Chromosomal instability in mammalian pre-implantation embryos: potential causes, detection methods, and clinical consequences. Cell Tissue Res 363: 201–225. 10.1007/s00441-015-2305-6 26590822 PMC5621482

[GR262774STAC11] Daughtry BL, Rosenkrantz JL, Lazar NH, Fei SS, Redmayne N, Torkenczy KA, Adey A, Yan M, Gao L, Park B, 2019 Single-cell sequencing of primate preimplantation embryos reveals chromosome elimination via cellular fragmentation and blastomere exclusion. Genome Res 29: 367–382. 10.1101/gr.239830.118 30683754 PMC6396419

[GR262774STAC12] Destouni A, Esteki MZ, Catteeuw M, Tšuiko O, Dimitriadou E, Smits K, Kurg A, Salumets A, Soom AV, Voet T, 2016 Zygotes segregate entire parental genomes in distinct blastomere lineages causing cleavage-stage chimerism and mixoploidy. Genome Res 26: 567–578. 10.1101/gr.200527.115 27197242 PMC4864459

[GR262774STAC13] Dobin A, Davis CA, Schlesinger F, Drenkow J, Zaleski C, Jha S, Batut P, Chaisson M, Gingeras TR. 2013 STAR: ultrafast universal RNA-seq aligner. Bioinformatics 29: 15–21. 10.1093/bioinformatics/bts635 23104886 PMC3530905

[GR262774STAC14] Dürrbaum M, Kuznetsova AY, Passerini V, Stingele S, Stoehr G, Storchová Z. 2014 Unique features of the transcriptional response to model aneuploidy in human cells. BMC Genomics 15: 139 10.1186/1471-2164-15-139 24548329 PMC3932016

[GR262774STAC15] Fan J, Lee H-O, Lee S, Ryu D, Lee S, Xue C, Kim SJ, Kim K, Barkas N, Park PJ, 2018 Linking transcriptional and genetic tumor heterogeneity through allele analysis of single-cell RNA-seq data. Genome Res 28: 1217–1227. 10.1101/gr.228080.117 29898899 PMC6071640

[GR262774STAC16] Fisher RA. 1925 Statistical methods for research workers, 11th ed. rev Oliver and Boyd, Edinburgh.

[GR262774STAC17] FitzPatrick DR. 2005 Transcriptional consequences of autosomal trisomy: primary gene dosage with complex downstream effects. Trends Genet 21: 249–253. 10.1016/j.tig.2005.02.012 15851056

[GR262774STAC18] Gleicher N, Metzger J, Croft G, Kushnir VA, Albertini DF, Barad DH. 2017 A single trophectoderm biopsy at blastocyst stage is mathematically unable to determine embryo ploidy accurately enough for clinical use. Reprod Biol Endocrinol 15: 33 10.1186/s12958-017-0251-8 28449669 PMC5408377

[GR262774STAC19] Golubovsky MD. 2003 Postzygotic diploidization of triploids as a source of unusual cases of mosaicism, chimerism and twinning. Hum Reprod 18: 236–242. 10.1093/humrep/deg060 12571155

[GR262774STAC20] Greco E, Minasi MG, Fiorentino F. 2015 Healthy babies after intrauterine transfer of mosaic aneuploid blastocysts. N Engl J Med 373: 2089–2090. 10.1056/NEJMc1500421 26581010

[GR262774STAC21] Griffiths JA, Scialdone A, Marioni JC. 2017 Mosaic autosomal aneuploidies are detectable from single-cell RNAseq data. BMC Genomics 18: 904 10.1186/s12864-017-4253-x 29178830 PMC5702132

[GR262774STAC22] Groff AF, Resetkova N, DiDomenico F, Sakkas D, Penzias A, Rinn JL, Eggan K. 2019 RNA-seq as a tool for evaluating human embryo competence. Genome Res 29: 1705–1718. 10.1101/gr.252981.119 31548358 PMC6771404

[GR262774STAC23] Haque A, Engel J, Teichmann SA, Lönnberg T. 2017 A practical guide to single-cell RNA-sequencing for biomedical research and clinical applications. Genome Med 9: 75 10.1186/s13073-017-0467-4 28821273 PMC5561556

[GR262774STAC24] Hassold T, Hunt P. 2001 To err (meiotically) is human: the genesis of human aneuploidy. Nat Rev Genet 2: 280–291. 10.1038/35066065 11283700

[GR262774STAC25] Home P, Kumar RP, Ganguly A, Saha B, Milano-Foster J, Bhattacharya B, Ray S, Gunewardena S, Paul A, Camper SA, 2017 Genetic redundancy of GATA factors in the extraembryonic trophoblast lineage ensures the progression of preimplantation and postimplantation mammalian development. Development 144: 876–888. 10.1242/dev.145318 28232602 PMC5374352

[GR262774STAC26] Kawai K, Harada T, Ishikawa T, Sugiyama R, Kawamura T, Yoshida A, Tsutsumi O, Ishino F, Kubota T, Kohda T. 2018 Parental age and gene expression profiles in individual human blastocysts. Sci Rep 8: 2380 10.1038/s41598-018-20614-8 29402920 PMC5799158

[GR262774STAC27] Kempf T, Eden M, Strelau J, Naguib M, Willenbockel C, Tongers J, Heineke J, Kotlarz D, Xu J, Molkentin J, 2006 The transforming growth factor-β superfamily member growth-differentiation factor-15 protects the heart from ischemia/reperfusion injury. Circ Res 98: 351–360. 10.1161/01.RES.0000202805.73038.48 16397141

[GR262774STAC28] Korotkevich G, Sukhov V, Sergushichev A. 2019 Fast gene set enrichment analysis. *bioRxiv* 10.1101/060012

[GR262774STAC29] Kort JD, McCoy RC, Demko Z, Lathi RB. 2018 Are blastocyst aneuploidy rates different between fertile and infertile populations? J Assist Reprod Genet 35: 403–408. 10.1007/s10815-017-1060-x 29063503 PMC5904052

[GR262774STAC30] Li H, Handsaker B, Wysoker A, Fennell T, Ruan J, Homer N, Marth G, Abecasis G, Durbin R, 1000 Genome Project Data Processing Subgroup. 2009 The Sequence Alignment/Map format and SAMtools. Bioinformatics 25: 2078–2079. 10.1093/bioinformatics/btp352 19505943 PMC2723002

[GR262774STAC31] Liberzon A, Birger C, Thorvaldsdóttir H, Ghandi M, Mesirov JP, Tamayo P. 2015 The molecular signatures database hallmark gene set collection. Cell Syst 1: 417–425. 10.1016/j.cels.2015.12.004 26771021 PMC4707969

[GR262774STAC32] Licciardi F, Lhakhang T, Kramer YG, Zhang Y, Heguy A, Tsirigos A. 2018 Human blastocysts of normal and abnormal karyotypes display distinct transcriptome profiles. Sci Rep 8: 14906 10.1038/s41598-018-33279-0 30297919 PMC6175822

[GR262774STAC33] Liu T, Zhang L, Joo D, Sun S-C. 2017 NF-κB signaling in inflammation. Signal Transduct Target Ther 2: 17023 10.1038/sigtrans.2017.23 29158945 PMC5661633

[GR262774STAC34] Loh P-R, Genovese G, Handsaker RE, Finucane HK, Reshef YA, Palamara PF, Birmann BM, Talkowski ME, Bakhoum SF, McCarroll SA, 2018 Insights into clonal haematopoiesis from 8,342 mosaic chromosomal alterations. Nature 559: 350–355. 10.1038/s41586-018-0321-x 29995854 PMC6054542

[GR262774STAC35] Macaulay IC, Haerty W, Kumar P, Li YI, Hu TX, Teng MJ, Goolam M, Saurat N, Coupland P, Shirley LM, 2015 G&T-seq: parallel sequencing of single-cell genomes and transcriptomes. Nat Methods 12: 519–522. 10.1038/nmeth.3370 25915121

[GR262774STAC36] McCoy RC. 2017 Mosaicism in preimplantation human embryos: when chromosomal abnormalities are the norm. Trends Genet 33: 448–463. 10.1016/j.tig.2017.04.001 28457629 PMC5484399

[GR262774STAC37] McCoy RC, Demko Z, Ryan A, Banjevic M, Hill M, Sigurjonsson S, Rabinowitz M, Fraser HB, Petrov DA. 2015a Common variants spanning *PLK4* are associated with mitotic-origin aneuploidy in human embryos. Science 348: 235–238. 10.1126/science.aaa3337 25859044 PMC5519344

[GR262774STAC38] McCoy RC, Demko ZP, Ryan A, Banjevic M, Hill M, Sigurjonsson S, Rabinowitz M, Petrov DA. 2015b Evidence of selection against complex mitotic-origin aneuploidy during preimplantation development. PLoS Genet 11: e1005601 10.1371/journal.pgen.1005601 26491874 PMC4619652

[GR262774STAC39] McKenna A, Hanna M, Banks E, Sivachenko A, Cibulskis K, Kernytsky A, Garimella K, Altshuler D, Gabriel S, Daly M, 2010 The Genome Analysis Toolkit: a MapReduce framework for analyzing next-generation DNA sequencing data. Genome Res 20: 1297–1303. 10.1101/gr.107524.110 20644199 PMC2928508

[GR262774STAC40] Munné S, Nakajima ST, Najmabadi S, Sauer MV, Angle MJ, Rivas JL, Mendieta LV, Macaso TM, Sawarkar S, Nadal A, 2020 First PGT-A using human *in vivo* blastocysts recovered by uterine lavage: comparison with matched IVF embryo controls. Hum Reprod 35: 70–80. 10.1093/humrep/dez242 31886877 PMC6993848

[GR262774STAC41] Nguyen NMP, Ge Z-J, Reddy R, Fahiminiya S, Sauthier P, Bagga R, Sahin FI, Mahadevan S, Osmond M, Breguet M, 2018 Causative mutations and mechanism of androgenetic hydatidiform moles. Am J Hum Genet 103: 740–751. 10.1016/j.ajhg.2018.10.007 30388401 PMC6218808

[GR262774STAC42] Ottolini CS, Newnham LJ, Capalbo A, Natesan SA, Joshi HA, Cimadomo D, Griffin DK, Sage K, Summers MC, Thornhill AR, 2015 Genome-wide maps of recombination and chromosome segregation in human oocytes and embryos show selection for maternal recombination rates. Nat Genet 47: 727–735. 10.1038/ng.3306 25985139 PMC4770575

[GR262774STAC43] Ottolini CS, Kitchen J, Xanthopoulou L, Gordon T, Summers MC, Handyside AH. 2017 Tripolar mitosis and partitioning of the genome arrests human preimplantation development *in vitro*. Sci Rep 7: 9744 10.1038/s41598-017-09693-1 28851957 PMC5575028

[GR262774STAC44] Petropoulos S, Edsgärd D, Reinius B, Deng Q, Panula SP, Codeluppi S, Plaza Reyes A, Linnarsson S, Sandberg R, Lanner F. 2016 Single-cell RNA-seq reveals lineage and X chromosome dynamics in human preimplantation embryos. Cell 165: 1012–1026. 10.1016/j.cell.2016.03.023 27062923 PMC4868821

[GR262774STAC045] R Core Team. 2019 R: a language and environment for statistical computing. R Foundation for Statistical Computing, Vienna. https://www.R-project.org/.

[GR262774STAC45] Rosenwaks Z, Handyside AH, Fiorentino F, Gleicher N, Paulson RJ, Schattman GL, Scott RT, Summers MC, Treff NR, Xu K. 2018 The pros and cons of preimplantation genetic testing for aneuploidy: clinical and laboratory perspectives. Fertil Steril 110: 353–361. 10.1016/j.fertnstert.2018.06.002 30098682

[GR262774STAC46] Sanchez-Ribas I, Diaz-Gimeno P, Sebastián-León P, Mercader A, Quiñonero A, Ballesteros A, Pellicer A, Domínguez F. 2019 Transcriptomic behavior of genes associated with chromosome 21 aneuploidies in early embryo development. Fertil Steril 111: 991–1001.e2. 10.1016/j.fertnstert.2019.01.023 30922649

[GR262774STAC47] Santaguida S, Richardson A, Iyer DR, M'Saad O, Zasadil L, Knouse KA, Wong YL, Rhind N, Desai A, Amon A. 2017 Chromosome mis-segregation generates cell-cycle-arrested cells with complex karyotypes that are eliminated by the immune system. Dev Cell 41: 638–651.e5. 10.1016/j.devcel.2017.05.022 28633018 PMC5536848

[GR262774STAC48] Sheltzer JM, Torres EM, Dunham MJ, Amon A. 2012 Transcriptional consequences of aneuploidy. Proc Natl Acad Sci 109: 12644–12649. 10.1073/pnas.1209227109 22802626 PMC3411958

[GR262774STAC49] Son M-Y, Choi H, Han Y-M, Cho YS. 2013 Unveiling the critical role of REX1 in the regulation of human stem cell pluripotency. Stem Cells 31: 2374–2387. 10.1002/stem.1509 23939908

[GR262774STAC50] Soneson C, Robinson MD. 2018 Bias, robustness and scalability in single-cell differential expression analysis. Nat Methods 15: 255–261. 10.1038/nmeth.4612 29481549

[GR262774STAC51] Stirparo GG, Boroviak T, Guo G, Nichols J, Smith A, Bertone P. 2018 Integrated analysis of single-cell embryo data yields a unified transcriptome signature for the human pre-implantation epiblast. Development 145: dev169672 10.1242/dev.169672 29361568 PMC5818005

[GR262774STAC52] Subramanian A, Tamayo P, Mootha VK, Mukherjee S, Ebert BL, Gillette MA, Paulovich A, Pomeroy SL, Golub TR, Lander ES, 2005 Gene set enrichment analysis: a knowledge-based approach for interpreting genome-wide expression profiles. Proc Natl Acad Sci 102: 15545–15550. 10.1073/pnas.0506580102 16199517 PMC1239896

[GR262774STAC53] Toutain J, Goutte-Gattat D, Horovitz J, Saura R. 2018 Confined placental mosaicism revisited: impact on pregnancy characteristics and outcome. PLoS One 13: e0195905 10.1371/journal.pone.0195905 29649318 PMC5897023

[GR262774STAC54] Trapnell C, Cacchiarelli D, Grimsby J, Pokharel P, Li S, Morse M, Lennon NJ, Livak KJ, Mikkelsen TS, Rinn JL. 2014 The dynamics and regulators of cell fate decisions are revealed by pseudotemporal ordering of single cells. Nat Biotechnol 32: 381–386. 10.1038/nbt.2859 24658644 PMC4122333

[GR262774STAC55] Tsai H-J, Nelliat AR, Choudhury MI, Kucharavy A, Bradford WD, Cook ME, Kim J, Mair DB, Sun SX, Schatz MC, 2019 Hypo-osmotic-like stress underlies general cellular defects of aneuploidy. Nature 570: 117–121. 10.1038/s41586-019-1187-2 31068692 PMC6583789

[GR262774STAC56] Tšuiko O, Catteeuw M, Zamani Esteki M, Destouni A, Bogado Pascottini O, Besenfelder U, Havlicek V, Smits K, Kurg A, Salumets A, 2017 Genome stability of bovine *in vivo*-conceived cleavage-stage embryos is higher compared to *in vitro*-produced embryos. Hum Reprod 32: 2348–2357. 10.1093/humrep/dex286 29040498

[GR262774STAC57] Vanneste E, Voet T, Le Caignec C, Ampe M, Konings P, Melotte C, Debrock S, Amyere M, Vikkula M, Schuit F, 2009 Chromosome instability is common in human cleavage-stage embryos. Nat Med 15: 577–583. 10.1038/nm.1924 19396175

[GR262774STAC58] Vázquez-Diez C, FitzHarris G. 2018 Causes and consequences of chromosome segregation error in preimplantation embryos. Reproduction 155: R63–R76. 10.1530/REP-17-0569 29109119

[GR262774STAC59] Vera-Rodriguez M, Chavez SL, Rubio C, Pera RAR, Simon C. 2015 Prediction model for aneuploidy in early human embryo development revealed by single-cell analysis. Nat Commun 6: 7601 10.1038/ncomms8601 26151134 PMC4506544

[GR262774STAC60] Weizman NF, Wyse BA, Antes R, Ibarrientos Z, Sangaralingam M, Motamedi G, Kuznyetsov V, Madjunkova S, Librach CL. 2019 Towards improving embryo prioritization: parallel next generation sequencing of DNA and RNA from a single trophectoderm biopsy. Sci Rep 9: 2853 10.1038/s41598-019-39111-7 30814554 PMC6393576

[GR262774STAC61] Zamani Esteki M, Viltrop T, Tšuiko O, Tiirats A, Koel M, Nõukas M, Žilina O, Teearu K, Marjonen H, Kahila H, 2019 In vitro fertilization does not increase the incidence of de novo copy number alterations in fetal and placental lineages. Nat Med 25: 1699–1705. 10.1038/s41591-019-0620-2 31686035

[GR262774STAC62] Zhou F, Wang R, Yuan P, Ren Y, Mao Y, Li R, Lian Y, Li J, Wen L, Yan L, 2019 Reconstituting the transcriptome and DNA methylome landscapes of human implantation. Nature 572: 660–664. 10.1038/s41586-019-1500-0 31435013

[GR262774STAC63] Zhu P, Guo H, Ren Y, Hou Y, Dong J, Li R, Lian Y, Fan X, Hu B, Gao Y, 2018 Single-cell DNA methylome sequencing of human preimplantation embryos. Nat Genet 50: 12–19. 10.1038/s41588-017-0007-6 29255258

