# Supplementary material for: Single-cell analysis of human embryos reveals diverse patterns of aneuploidy and mosaicism
Source: Genome Res. 2020 Jun;30(6):814–25. doi: 10.1101/gr.262774.120 (PMC7370883; doi:10.1101/gr.262774.120)
Supplement: Supplemental Material [file supp_30_6_814__index.html]

Single-cell analysis of human embryos reveals diverse patterns of aneuploidy and mosaicism — Single-cell analysis of human embryos reveals diverse patterns of aneuploidy and mosaicism — Supplemental Material 

# Single-cell analysis of human embryos reveals diverse patterns of aneuploidy and mosaicism

## Supplemental Material

- Supplemental\_Code.zip
- Supplemental\_Figures.pdf
- Supplemental\_Tables.pdf
