# Supplementary material for: Single-cell analysis of human embryos reveals diverse patterns of aneuploidy and mosaicism
Source: Genome Res. 2020 Jun;30(6):814–25. doi: 10.1101/gr.262774.120 (PMC7370883; doi:10.1101/gr.262774.120)
Supplement: Supplemental Material [file supp_gr.262774.120_Supplemental_Figures.pdf]

Supplemental figures

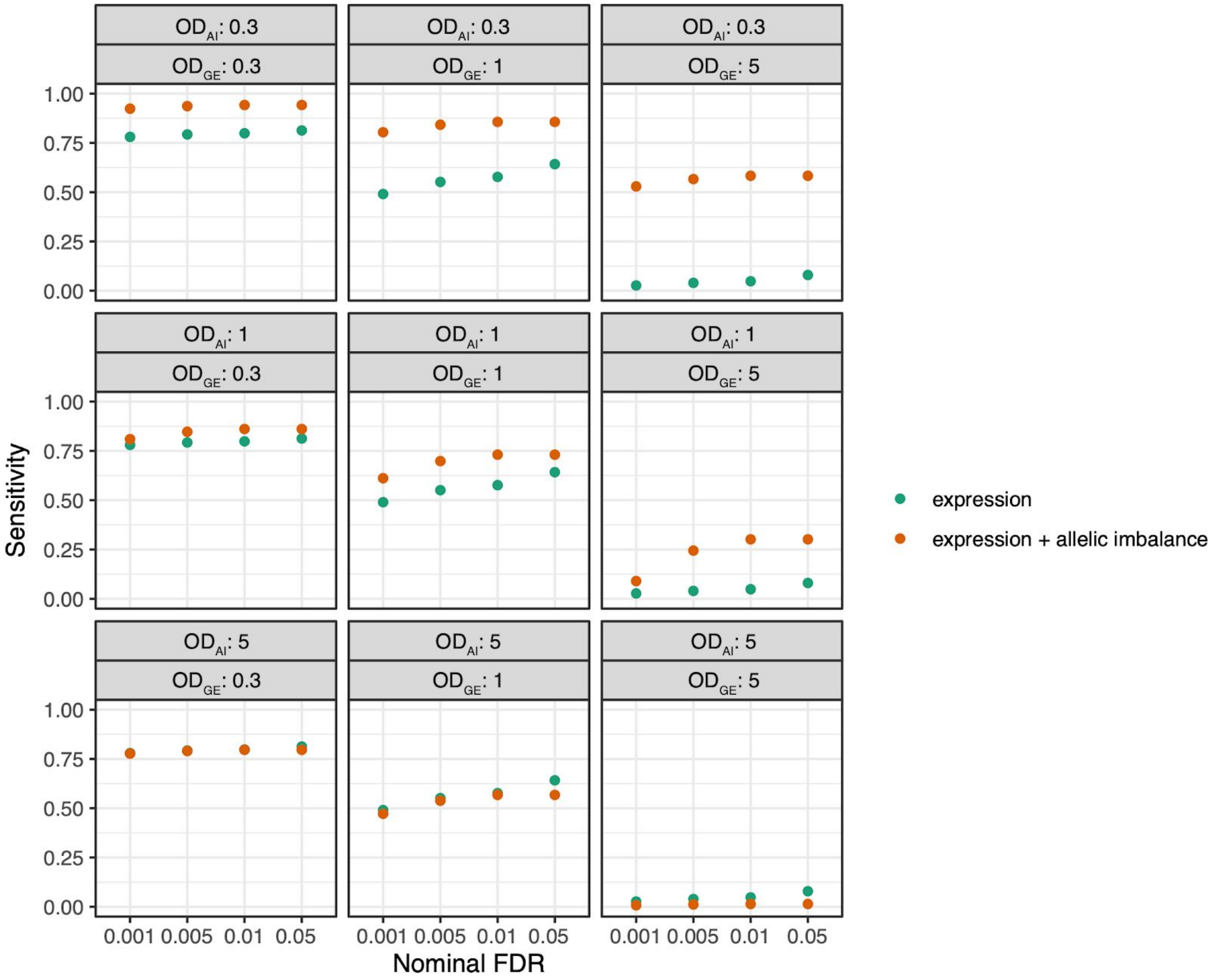

Fig. S1. Sensitivity for detecting aneuploidy in simulated data based on signatures of total gene expression alteration with and without integrating signatures of allelic imbalance. Panels represent different combinations of overdispersion parameters used to scale the variance in allelic imbalance (OD_{AI}) and gene expression (OD_{GE}) relative to the variance observed in the actual data (center panel).

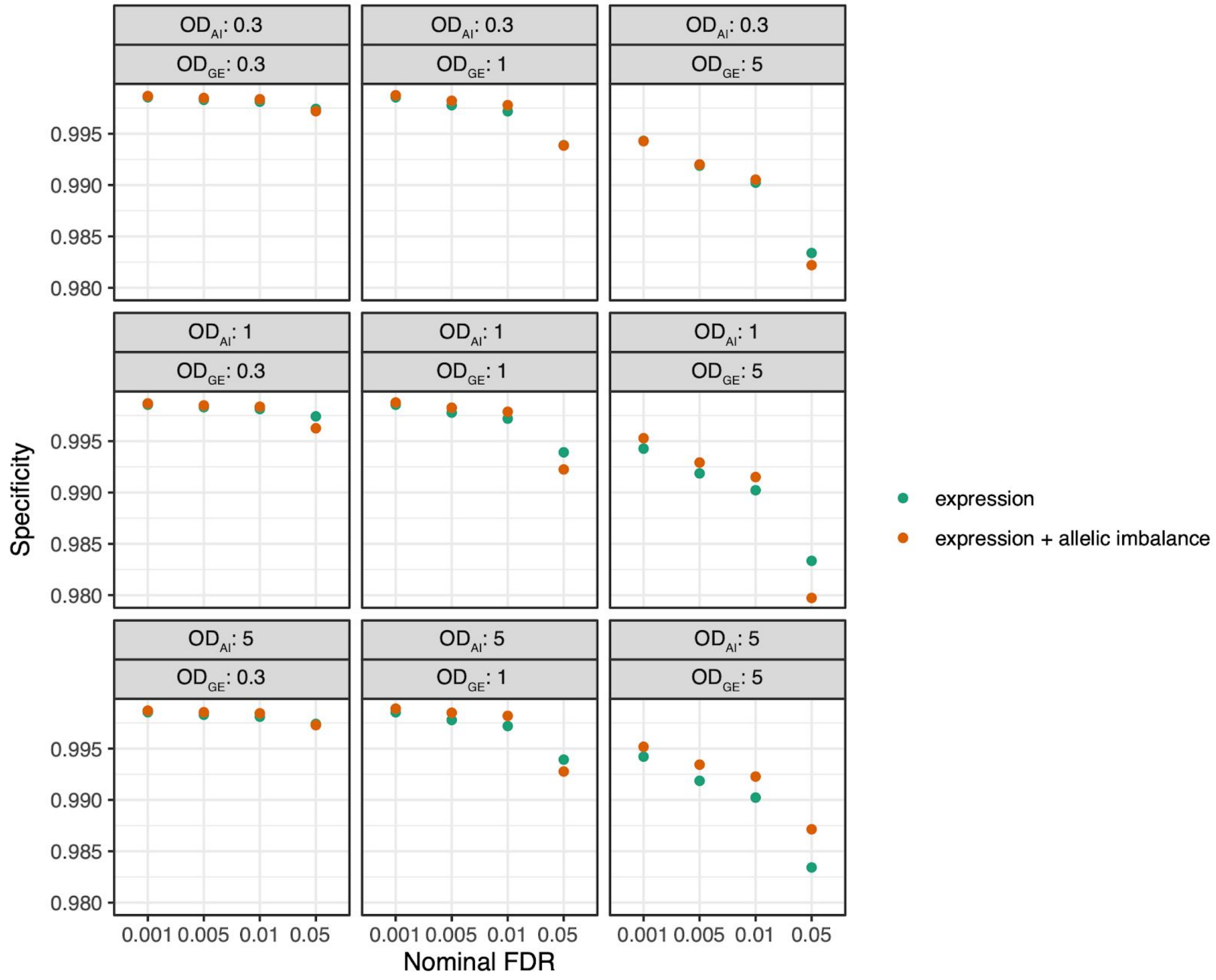

Fig. S2. Specificity for detecting aneuploidy in simulated data based on signatures of total gene expression alteration with and without integrating signatures of allelic imbalance. Panels represent different combinations of overdispersion parameters used to scale the variance in allelic imbalance (OD_{AI}) and gene expression (OD_{GE}) relative to the variance observed in the actual data (center panel).

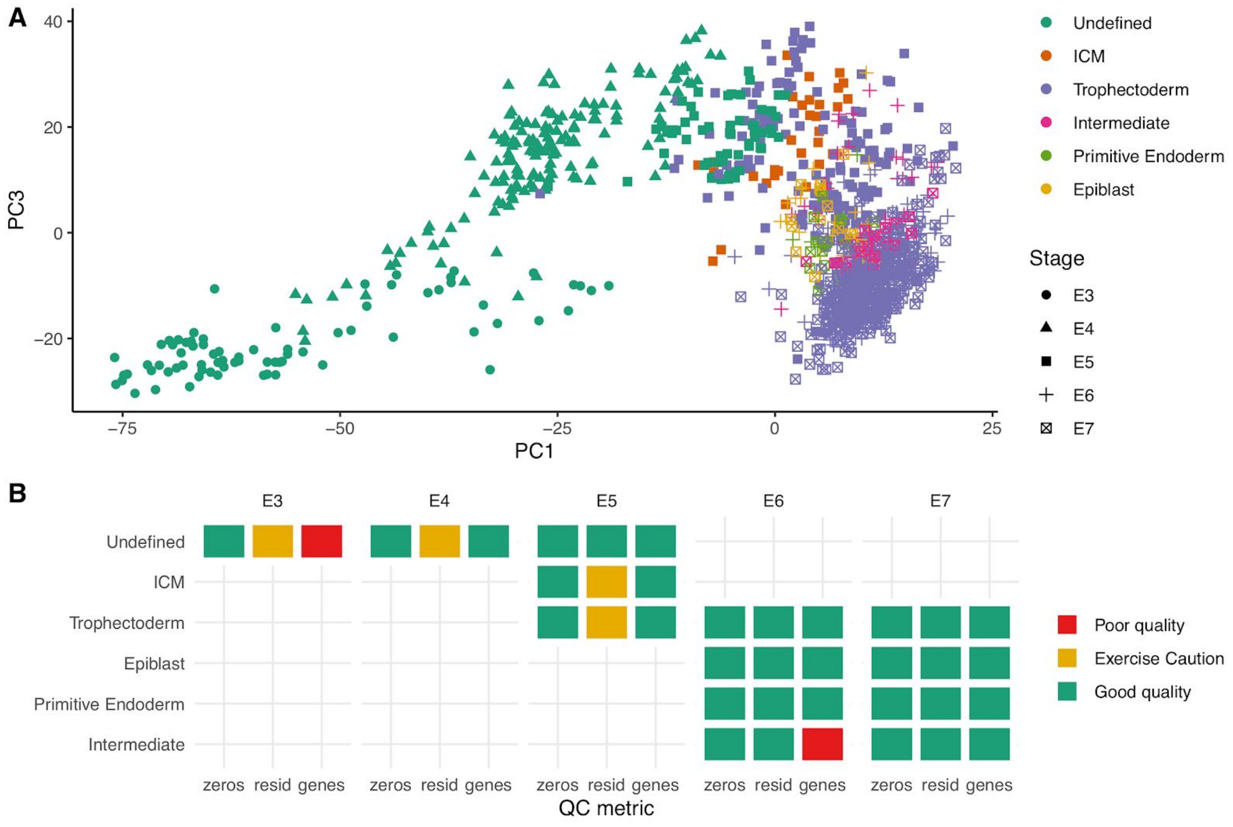

Fig. S3. Quality-control pre-processing of single-cell expression data. **A.** Principal component (PC) diagram of single-cell gene expression annotated with stage and cell type annotated based on Stirparo et al. (2018). These categories were used as grouping factors for *scploid*. PC1 and PC3 are depicted, as they best capture variation in stage and cell type, respectively. **B.** Quality control metrics computed by *scploid* for each stage/cell-type group. Groups failing a quality-control test (denoted as “Poor quality”) were excluded from subsequent analysis.

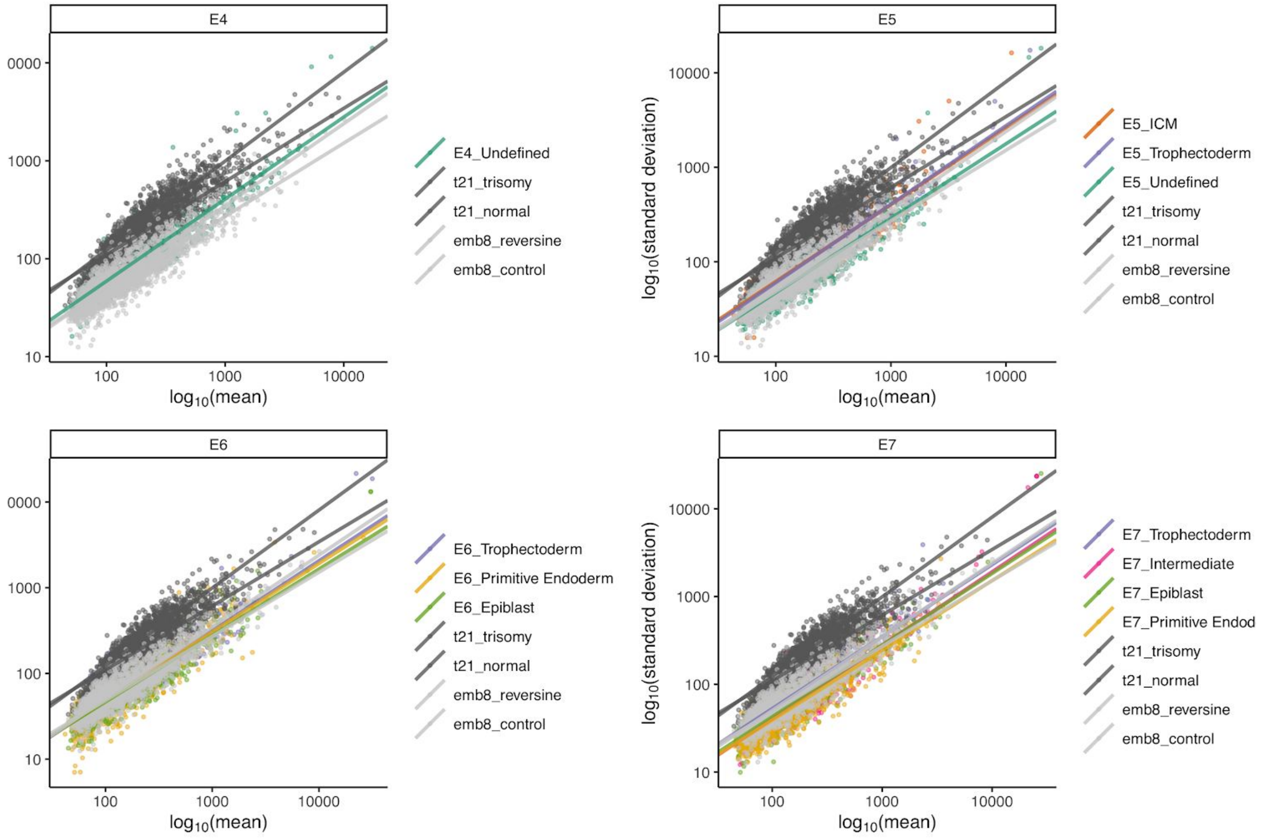

Fig. S4. Relationship between gene expression mean and standard deviation. Data from Petropoulos et al. (2016) are stratified by embryonic stage (days post-fertilization) and compared to mouse 8-cell embryo (emb8) and human trisomy 21 neuron (t21) data. These comparison data were previously used for *scploid* benchmarking by Griffiths et al. (2017) with good and poor performance, respectively.

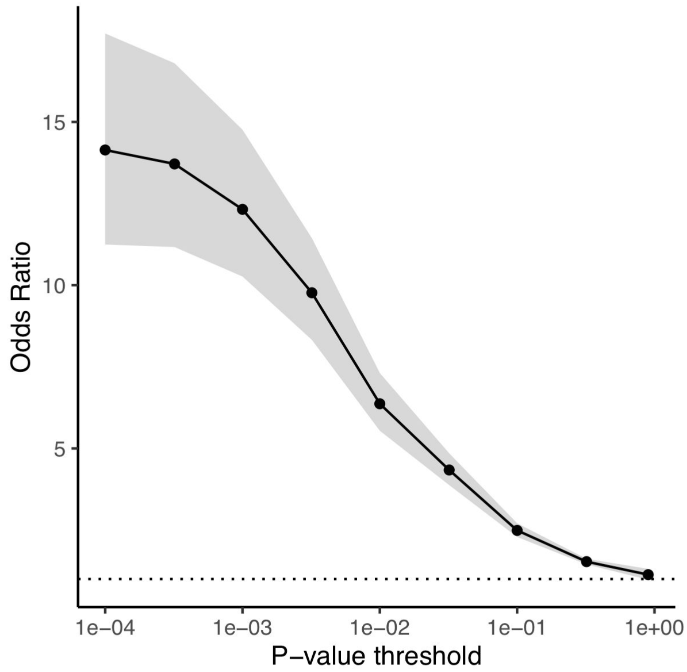

Fig. S5. Concordance between complementary signatures of aneuploidy. At increasingly stringent thresholds, we observe increasing overlap between chromosomes called aneuploid based on evidence of expression alteration and allelic imbalance. Shaded region indicates 95% confidence interval.

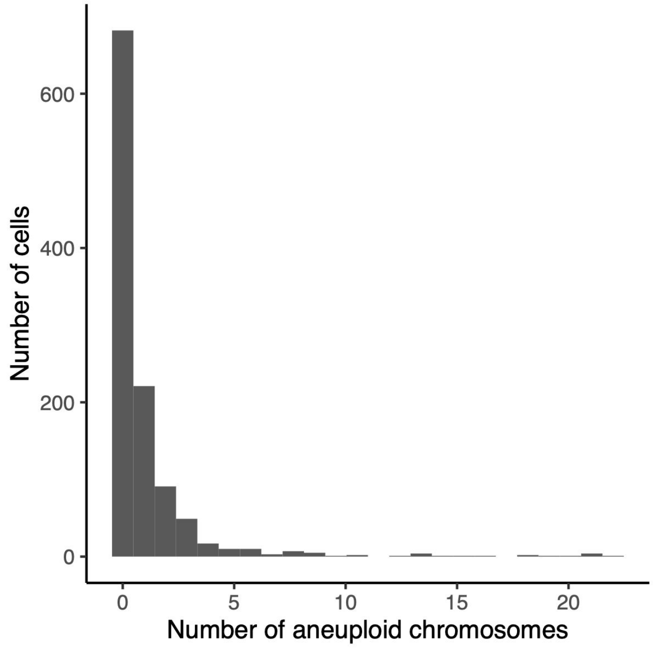

Fig. S6. Histogram of the inferred number of aneuploid chromosomes per cell (at 1% FDR).

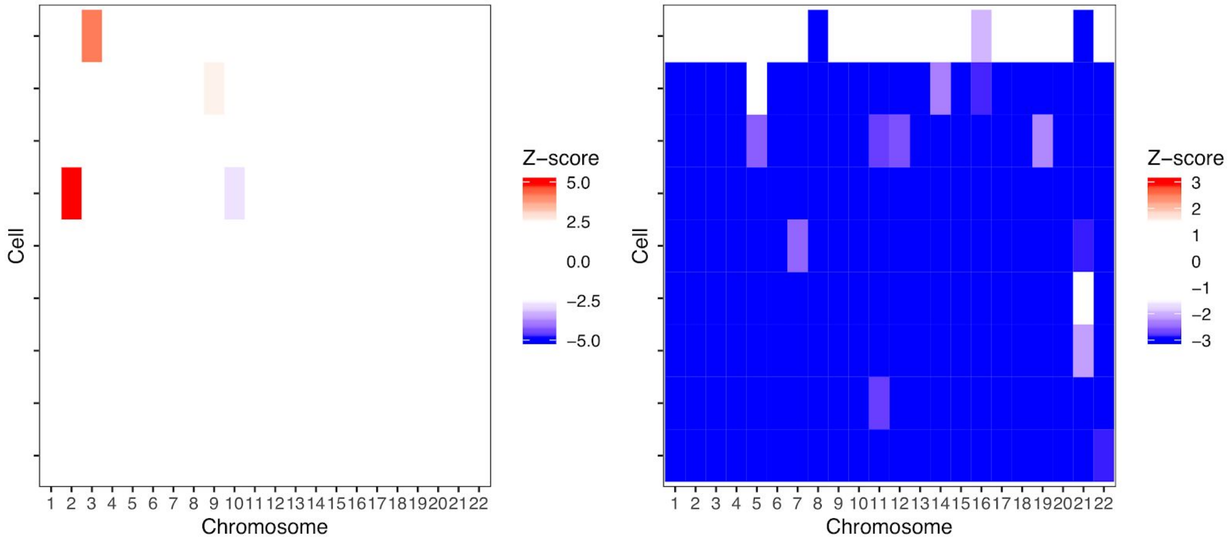

Fig. S7. Karyotype-wide abnormalities such as haploidy/near-haploidy are undetectable for embryo E7.5 based on expression signatures (left panel), but are evident based on widespread monoallelic expression (right panel). One cell (top row) exhibits mostly biallelic expression with only two monosomic chromosomes such that we classify the embryo as mosaic near-haploid.

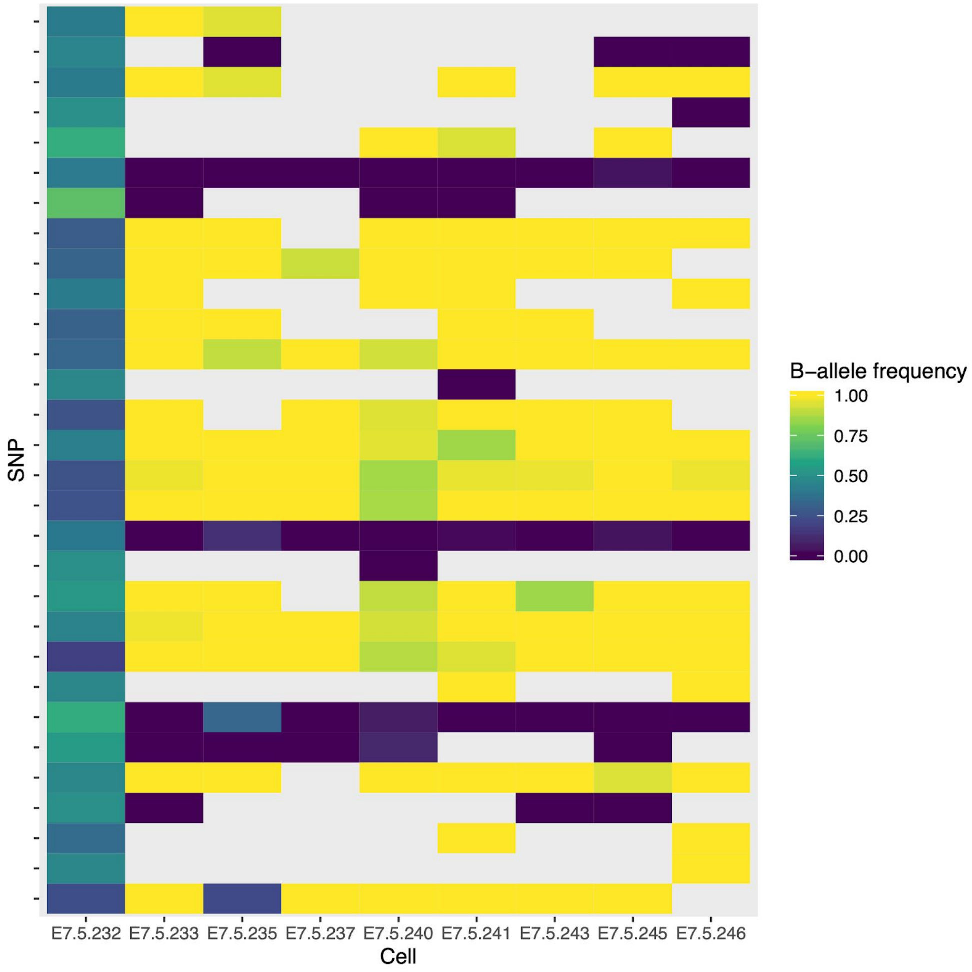

Fig. S8. Patterns of allelic imbalance in near-haploid cells of embryo 7.5. At biallelic SNPs discovered in cell E7.5.232 (≥5 reads supporting each allele), we tabulated the ratio of reads supporting the alternative allele to total reads (i.e., "B-allele frequency") for all other cells from the same embryo. With few exceptions, these reads consistently supported a single allele, consistent with a common parental origin. Cells with fewer than 9 total reads at a given SNP were omitted from our analysis and are depicted in gray.

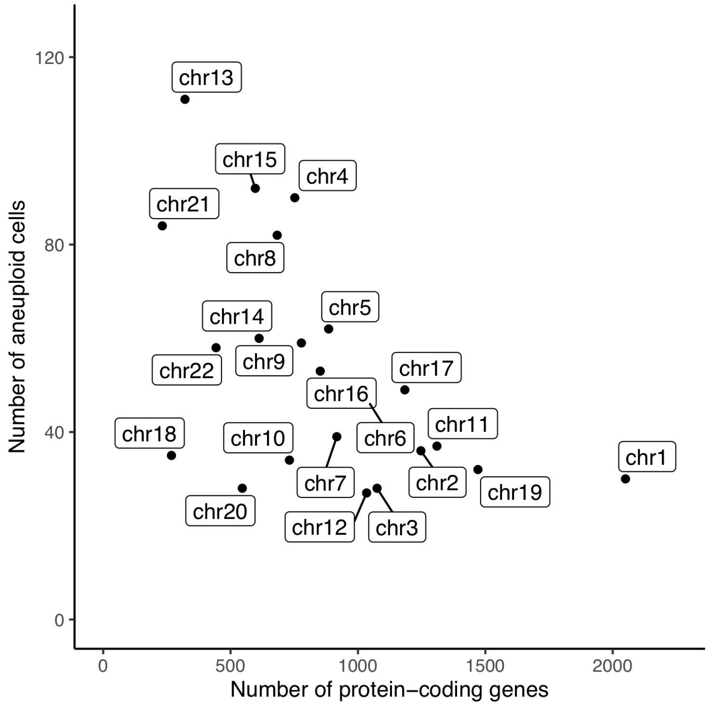

Fig. S9. Negative correlation between chromosome-specific aneuploidy rates and number of protein-coding genes per chromosome (Pearson's r = -0.546, p = 8.64×10^{-3}). Differences in aneuploidy rates among chromosomes were not significant, however, after accounting for the correlation among chromosomes within cells within embryos (χ^2(df = 21, n = 24,530) = 29.0, p = 0.114).

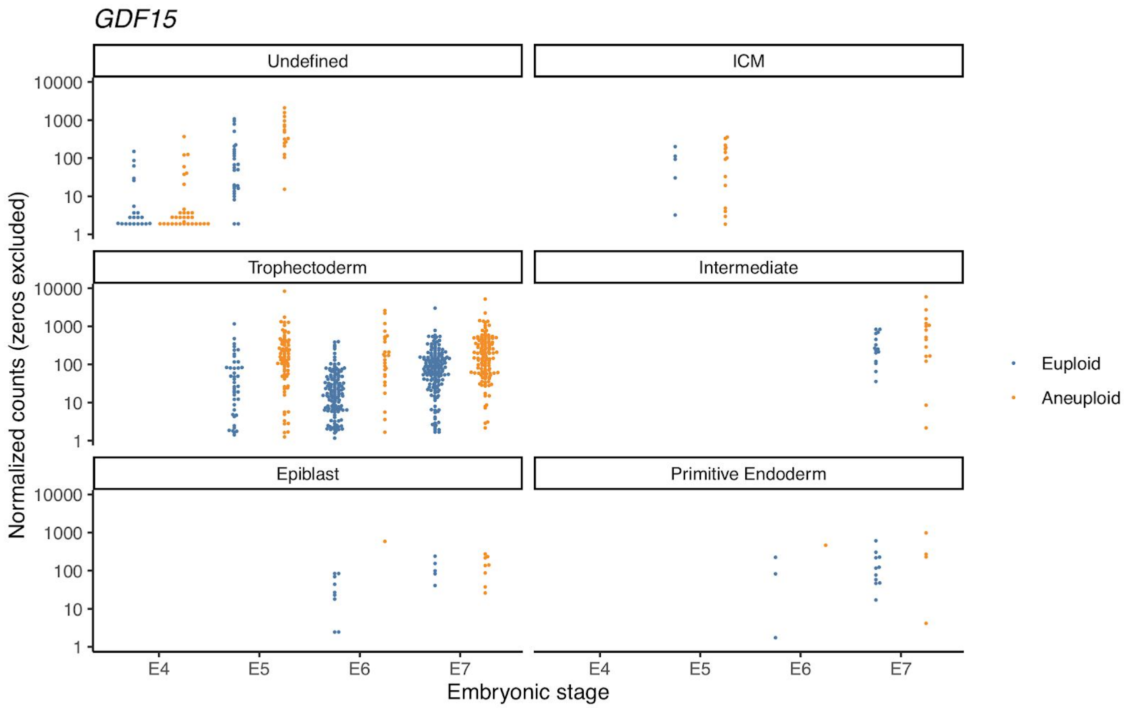

Fig. S10. Upregulation of *Growth/differentiation factor 15* (*GDF15*) in aneuploid compared to euploid cells.

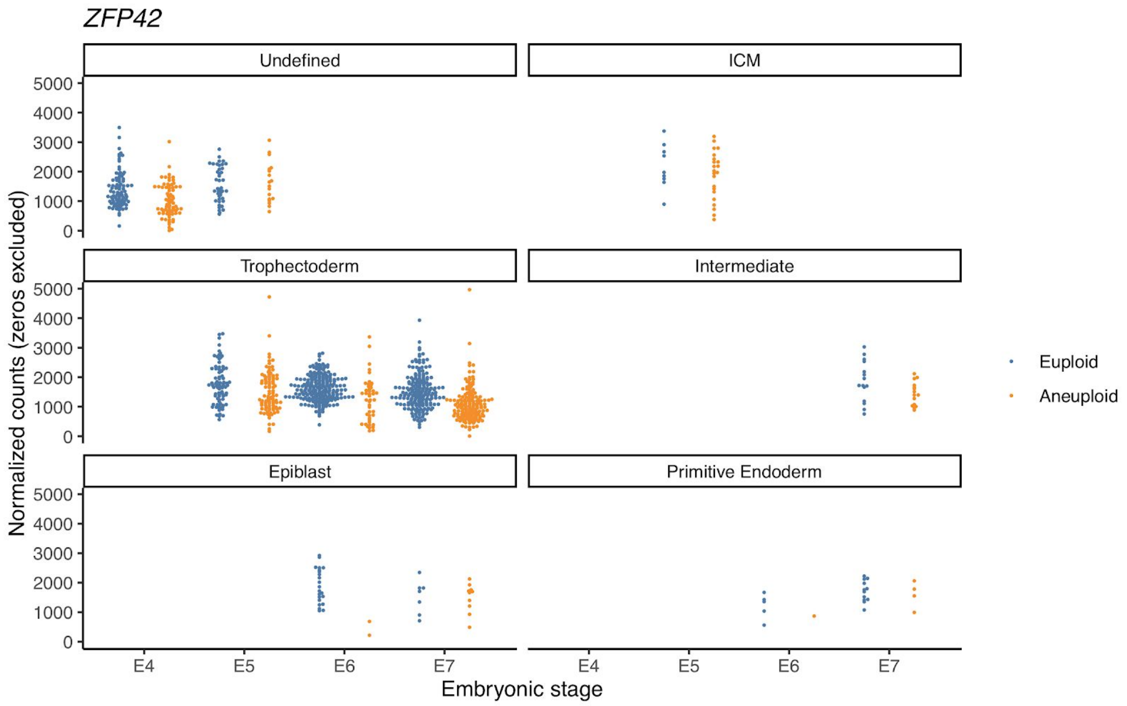

Fig. S11. Downregulation of *Zinc finger protein 42 homolog* (*ZFP42*) in aneuploid compared to euploid cells.

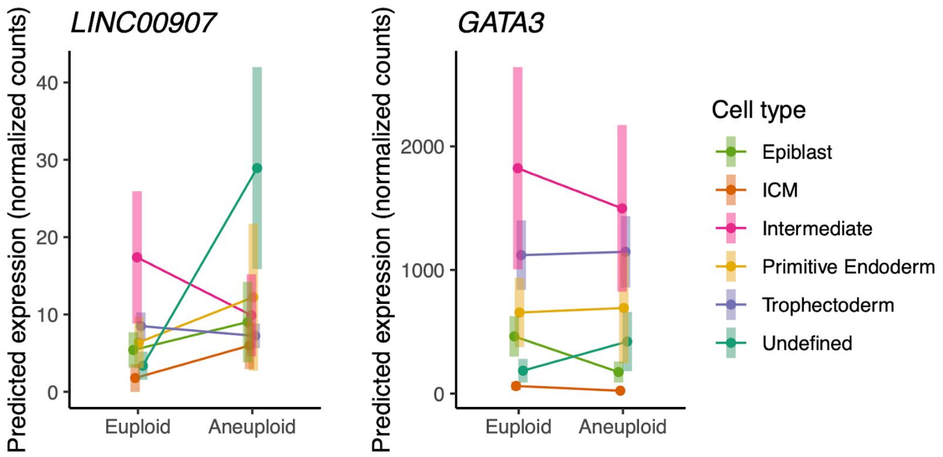

Fig. S12. Cell-type-specific responses to aneuploidy for genes *LINC00907* (left panel) and *GATA3* (right panel). Error bars indicate 95% confidence intervals. For *LINC00907*, only undifferentiated (undefined) cells exhibited a significant response to aneuploidy. For *GATA3*, the inner cell mass (ICM), epiblast, and undifferentiated cell-types displayed a significant aneuploidy response.

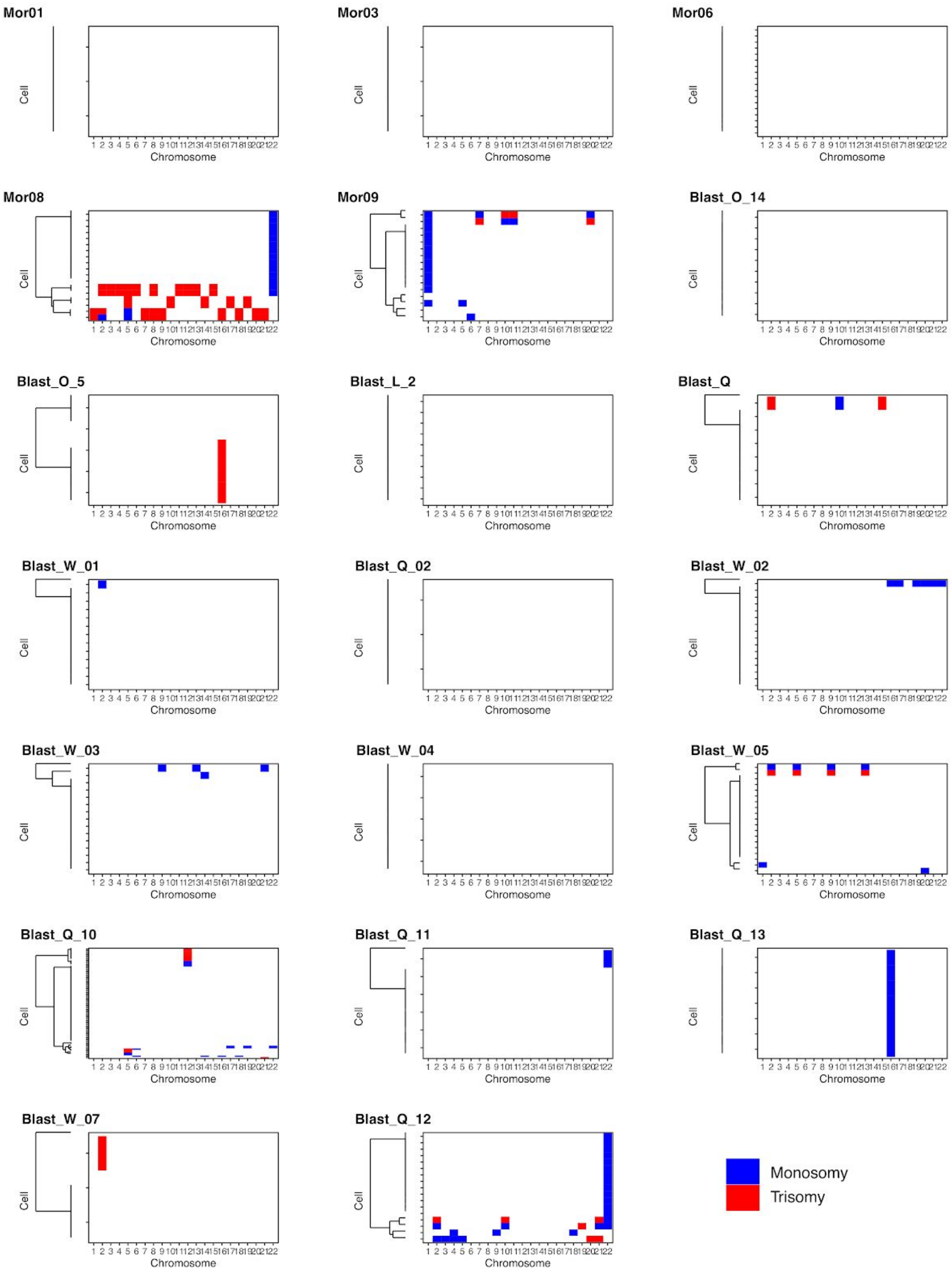

Fig. S13. Heatmaps of published aneuploidy calls based on PBAT scDNA-seq from Zhu et al. (2018b).
