# Supplementary material for: Single-cell analysis of human embryos reveals diverse patterns of aneuploidy and mosaicism
Source: Genome Res. 2020 Jun;30(6):814–25. doi: 10.1101/gr.262774.120 (PMC7370883; doi:10.1101/gr.262774.120)
Supplement: Supplemental Material [file supp_gr.262774.120_Supplemental_Tables.pdf]

Supplemental tables

	Nominal FDR	Sensitivity	Specificity	Precision	FDR	F1 Score
**expression**	0.001	0.490	0.9985	0.944	0.0559	0.645
**expression + allelic imbalance**	0.001	0.612	0.9988	0.961	0.0386	0.747
**expression**	0.005	0.551	0.9978	0.926	0.0743	0.691
**expression + allelic imbalance**	0.005	0.698	0.9983	0.953	0.0474	0.806
**expression**	0.010	0.576	0.9972	0.912	0.0884	0.706
**expression + allelic imbalance**	0.010	0.731	0.9979	0.945	0.0550	0.824
**expression**	0.050	0.642	0.9939	0.841	0.1587	0.728
**expression + allelic imbalance**	0.050	0.731	0.9922	0.826	0.1744	0.775

Table S1. Performance metrics for simulations using varying thresholds for the nominal false discovery rate. We compare results using the gene expression signature alone to using combined signatures of gene expression alteration and allelic imbalance. Results of simulations using an overdispersion factor of 1 (i.e., equal to the original data) are provided here. Results for simulations with additional overdispersion factors are provided in Fig. S1 and S2.

	E4	E5	E6	E7	Total
**Embryos**	16	23	18	17	74
**Cells**	160	262	290	403	1115
Undefined	160	60			220
Inner cell mass		31			31
Trophectoderm		171	260	340	771
Intermediate				30	30
Epiblast			24	17	41
Primitive endoderm			6	16	22

Table S2. Counts and embryos and cells from Petropoulos et al. (2016) that were analyzed in this study, stratified by stage (days post-fertilization). Cells are further stratified by cell type.

Symbol	β	SE (β)	P (β)	AME	SE (AME)	P (AME)
GDF15	1.118	0.144	6.6×10^{-15}	142.06	58.40	0.015
ZFP42	-0.262	0.037	1.6×10^{-12}	-375.97	59.34	2.4×10^{-10}
POLR2E	-0.214	0.031	6.3×10^{-12}	-455.35	75.43	1.6×10^{-9}
PRPF31	-0.314	0.047	2.6×10^{-11}	-87.83	14.13	5.2×10^{-10}
NCL	-0.156	0.024	9.1×10^{-11}	-1102.83	174.51	2.6×10^{-10}
HNRNPC	-0.168	0.027	2.4×10^{-10}	-1170.02	189.51	6.7×10^{-10}
TUBA1B	-0.254	0.041	4.9×10^{-10}	-1587.99	331.07	1.6×10^{-6}
AHSA1	-0.209	0.035	2.4×10^{-9}	-328.67	58.29	1.7×10^{-8}
VDAC1	-0.185	0.031	2.4×10^{-9}	-345.92	74.55	3.5×10^{-6}
HMOX2	-0.298	0.050	2.5×10^{-9}	-100.93	19.75	3.2×10^{-7}
AAMP	-0.236	0.040	3.4×10^{-9}	-327.56	59.94	4.6×10^{-8}
AP000459.7	0.433	0.073	3.5×10^{-9}	1.85	0.39	2.1×10^{-6}
SEH1L	-0.216	0.037	3.5×10^{-9}	-178.47	30.49	4.8×10^{-9}
GPI	-0.232	0.039	3.9×10^{-9}	-344.82	76.69	6.9×10^{-6}
SLC4A1	0.487	0.084	5.5×10^{-9}	4.06	0.79	2.6×10^{-7}
SSH3	-0.773	0.134	7.1×10^{-9}	-20.73	5.80	3.4×10^{-4}
TBRG4	-0.263	0.046	9.1×10^{-9}	-222.93	41.54	8.0×10^{-8}
TRAP1	-0.269	0.047	1.1×10^{-8}	-239.05	52.04	4.4×10^{-6}
TIMM44	-0.240	0.042	1.2×10^{-8}	-159.01	29.99	1.1×10^{-7}
HARS	-0.248	0.044	1.3×10^{-8}	-190.60	36.85	2.3×10^{-7}

Table S3. Top twenty associations from analysis of differential expression comparing euploid and aneuploid cells. Regression coefficients (β) and average marginal effects (AME) are reported, along with corresponding standard errors and p-values. Positive coefficients indicate upregulation in aneuploid cells relative to euploid cells, while negative coefficients indicate downregulation. Note that the β's are the embryo and cell-type-specific log incidence rate ratios, while the AMEs are the differences in the incidence rates averaged over all embryos and cell-types in our model.
